# Bioinspired Bola-Type Peptide Dendrimers Inhibit Proliferation and Invasiveness of Glioblastoma Cells in a Manner Dependent on Their Structure and Amphipathic Properties

**DOI:** 10.3390/pharmaceutics12111106

**Published:** 2020-11-18

**Authors:** Maciej Cieślak, Damian Ryszawy, Maciej Pudełek, Magdalena Urbanowicz, Maja Morawiak, Olga Staszewska-Krajewska, Jarosław Czyż, Zofia Urbańczyk-Lipkowska

**Affiliations:** 1 Institute of Organic Chemistry, Polish Academy of Sciences, 01-224 Warsaw, Poland; maciej.cieslak@icho.edu.pl (M.C.); m.urbanowicz@nil.gov.pl (M.U.); maja.morawiak@icho.edu.pl (M.M.); olga.krajewska@icho.edu.pl (O.S.-K.); 2 Department of Cell Biology, Faculty of Biochemistry, Biophysics and Biotechnology, Jagiellonian University, 30-387 Kraków, Poland; damian.ryszawy@uj.edu.pl (D.R.); maciej.pudelek@student.uj.edu.pl (M.P.)

**Keywords:** glioblastoma, bioinspired, dendrons, dendrimers, bola-structure, chemotherapy, malignancy

## Abstract

(1) Background: Natural peptides supporting the innate immune system studied at the functional and mechanistic level are a rich source of innovative compounds for application in human therapy. Increasing evidence indicates that apart from antimicrobial activity, some of them exhibit selective cytotoxicity towards tumor cells. Their cationic, amphipathic structure enables interactions with the negatively-charged membranes of microbial or malignant cells. It can be modeled in 3D by application of dendrimer chemistry. (2) Methods: Here we presented design principles, synthesis and bioactivity of branched peptides constructed from ornithine (Orn) assembled as proline (Pro)- or histidine (His)-rich dendrons and dendrimers of the bola structure. The impact of the structure and amphipathic properties of dendrons/dendrimers on two glioblastoma cell lines U87 and T98G was studied with the application of proliferation, apoptosis and cell migration assays. Cell morphology/cytoskeleton architecture was visualized by immunofluorescence microscopy. (3) Results: Dimerization of dendrons into bola dendrimers enhanced their bioactivity. Pro- and His-functionalized bola dendrimers displayed cytostatic activity, even though differences in the responsiveness of U87 and T98G cells to these compounds indicate that their bioactivity depends not only on multiple positive charge and amphipathic structure but also on cellular phenotype. (4) Conclusion: Ornithine dendrons/dendrimers represent a group of promising anti-tumor agents and the potential tools to study interrelations between drug bioactivity, its chemical properties and tumor cells’ phenotype.

## 1. Introduction

Numerous recent studies point to an increasing role of peptides as potential therapeutics for the treatment of a broad range of diseases. Since the introduction of the Merrifield’s solid state methods and recombinant technology to produce therapeutic peptides their production was advanced significantly. However, due to the size-related poor transport and instability in body fluids, therapeutic peptides and proteins are predominantly administered by either intravenous or subcutaneous injection, which raises the problem of bioavailability. The rapidly growing field of nanotechnology addresses some of these problems, mainly focusing on the development of appropriate nanocarriers for peptide therapeutics [1,2]. Examples of such peptide compounds include active per se low and high molecular weight peptides and proteins (e.g., insulin, growth hormone), targeting and transporting peptides [3,4], or peptide-drug bioconjugates [5,6]. Of great importance is the research towards the application of natural antimicrobial peptides (AMP’s) and their analogs, as they are capable of penetrating cell membranes and delivering various cargos to specific intracellular compartments [7,8,9]. AMP’s display amphipathic, cationic structure and rich functional characteristics featuring antimicrobial, antifungal, anticancer, etc., activity often associated with primary interactions with cell membranes [10]. The last competence might be of particular importance for brain tumor treatment where the poor penetration of the blood–brain barrier (BBB) and the poor internalization of the drugs by tumor cells preclude effective therapy.

Glioblastoma is one of the most aggressive and less curable human malignancies due to unpredictable proliferation and high invasiveness. Since the efficacy of the conventional glioblastoma chemotherapies (e.g., temozolomide- and carmustine-based chemotherapy combined with surgery and radiotherapy) is limited, various types of chemical entities were proposed for alternative treatments. Recently, the application of cationic host defense peptides was rationalized for glioblastoma treatment by the studies of Riedl et al., who selectively targeted negatively charged phosphatidylserine molecules located on the external leaflet of the membranes [11] of melanoma, glioblastoma and a rhabdomyosarcoma cells as well as prostate and renal cancer cells by analogs of human Lactoferricin [12,13]. Following these findings, efficacy of several structurally different AMP’s alone [14,15,16] or as hybrid materials [5,17,18,19,20] was tested against glioblastoma cells and showed their substantial activity along with structure-dependent versatility of mechanism of action [21,22,23].

Since the production of AMP’s in large quantities is still problematic, we designed several series of AMP’ mimetics in the form of amphiphilic branched peptides (dendrimers or dendrons) to show their significant activity against bacteria [24,25], fungi [26,27,28], melanoma [29] and glioblastoma cells [30]. The branched structure facilitated the localization/exposition of multiple side chains of amino acids and other groups on their molecular surface. Apparently, they can arrange themselves in a 3D fashion meaningful for the molecular recognition and biological activity [31].

Considering the structural attributes of dendrimeric compounds, i.e., the location of multiple ligands and positive charges in a dendrimer “tree”, cationic AMP’s with multiple copies of certain amino acids are the most interesting for synthetic design. Recently, anticancer properties were documented for two series of histidine-rich natural peptides: isolated from human saliva histatins [32,33,34] and Tilapia piscidin 4 (TP4) peptide that beyond the antimicrobial [35] and wide anticancer properties [36,37] expressed also the activity against glioblastoma cells. The above peptides as well as human peptide RL-37, have 3–4 copies of histidine located at the N-terminus. They adopt random conformation in hydrophilic and an α-helical conformation in organic solutions. The second group includes AMP’s rich in proline, such as a porcine cathelicidin PR39 peptide that suppresses migration and invasion of breast cancer cells [38] and mammalian host defense peptides belonging to the cathelicidin group that are carrying multiple prolines inside of the linear sequence. Among them goncocin, apidaecin, and drosocin [39], which are mainly excellent antimicrobials, can cross the blood–brain barrier and selectively reach the brain target [40,41].

Considering the above findings, we designed and synthesized a series of dendrimers with a bola-type structure constructed from two ornithine-based first generation monomeric dendrons connected with a linear spacer. Dendrimer branches were terminated with multiple copies of two amino acids abundant in human AMP’s that are active against malignant cells—histidine (His) or proline (Pro). To assess the significance of (i) bola-type dimerization, (ii) presence of positive charges and (iii) possible molecule biodegradation for their biological performance, two approaches were taken. First, monomeric dendrons assembled from ornithine (Orn) and functionalized with histidine (His), nitro-arginine (Arg-NO_2_) or proline (Pro) were synthesized. Second, bola dimers were assembled from monomeric dendrons and linkers via chemically stable amide or biodegradable ester bonds. Within this series, His-decorated bola dendrimers are cationic and amphiphilic whereas Pro-decorated bola-dimers are neutral. Then, the biological activity of dendrons and bola dendrimers was assessed by the analyses of their effects on the proliferation, viability and invasiveness of glioblastoma cells. Our data provide insight into the complexity of the determinants of dendrons/dendrimers bioactivity with the possible role of tumor-specific cell reactivity to these compounds.

## 2. Materials and Methods

### 2.1. General Procedures

All solvents and reagents were of analytical grade and were used without further purification. Coupling reagents *N*,*N*′-Dicyclohexylcarbodiimide (DCC), *N*-Hydroxysuccinimide (HOSu), *N*-Hydroxybenzotriazol (HOBt), HCl-saturated AcOEt, 4,7,10-trioxa-1,13-tridecanediamine, *O*,*O*′-bis(2-aminoethyl)polyethylene glycol as well as all solvents were purchased from Sigma (Steinheim, Germany).

Mass spectra were recorded with a Mariner ESI time-of-flight mass spectrometer (PerSeptive Biosystems, Foster City, CA, USA) for the samples prepared in MeOH. The ^1^H-NMR and ^13^C-NMR spectra were recorded using a Varian VNMRS 500/125 MHz and 600/150 MHz spectrometers, respectively (Varian, Inc., acquired by Agilent Technologies, Palo Alto, CA, USA) at 500/125 or 400/100 MHz, respectively, using deuterated solvents and TMS as an internal standard. Chemical shifts are reported as δ values in parts per million, and coupling constants are given in hertz. The optical rotations were measured with a JASCO J-1020 digital polarimeter (Ishikawa-machi, Hachioji, Tokyo, Japan). Melting points were recorded on a Köfler hot-stage apparatus (Wagner & Munz, München, Germany) and are uncorrected. Thin layer chromatography (TLC) was performed on aluminum sheets with silica gel 60 F254 from Merck (Darmstadt, Germany). Column chromatography (CC) was carried out using silica gel (230–400 mesh) from Merck or Sephadex LH20 (Darmstadt, Germany or Biosciences, Upsala, Sweden). The TLC spots were visualized by treatment with 1% EtOH solution of ninhydrin and heating.

HPLC analysis for bola was performed with a Knauer HPLC system equipped with a dual wavelength (λ) absorbance detector at 214 and 280 nm (KnauerBerlin, Berlin, Germany). The crude products were purified by preparative HPLC using a C_18_ column, (Bionacom Velocity C-18-LPH) 250 × 212 mm, particle size 10μm, pore diameter of 200 Å, followed by processing by analytical HPLC column (Luna LC-Column, C-8(2)) 150 × 46 mm, particle size 3 μm, pore diameter 100 Å (Bionacom LTD, Coventry, England). The mobile phase consisted of a gradient from 5 to 95% MeOH/H_2_O, 0.05% HCl, at a flow rate of 2.0 mL/min (analytical) or 9 mL/min (preparative).

### 2.2. Cell Culture

Human T98G (ATCC, CRL-1690) and U87 (ATCC, HTB-14) glioblastoma cells were cultured in 25 cm^2^ tissue culture-treated flasks (Eppendorf AG, Hamburg, Germany) in high glucose (4500 mg/L) DMEM medium (Sigma), supplemented with 10% FBS (Gibco, Thermo Fisher Scientific, Waltham, MA, USA) and 1% Antibiotic Antimycotic Solution (Sigma, St. Louis, MO, USA), in standard conditions (5% CO_2_, 37 °C). For experiments, cells were harvested with TrypLE (Invitrogen/Thermo Fisher Scientific, Waltham, MA, USA) after washing with Ca^2+^/Mg^2+^-free HBSS (Corning, NY, USA), re-suspended in culture medium, counted in Z2 particle counter (Beckman Coulter, Indianapolis, IN, USA) and seeded into multi-well flasks. Examined dendrons and bola dendrimers were dissolved in pure DMSO (Sigma) and applied in cell culture at concentration range 10–200 µM in indicated time steps (24–96 h). In control samples, the cells were treated with vehicle (DMSO) administered at the concentrations equivalent to the highest compound concentrations.

### 2.3. Proliferation and Apoptosis Assays

Cells were seeded into 12-well culture plates (Eppendorf) at the density of 2 × 10^4^/cm^2^, cultivated for 24 h before the addition of dendrons and bola dendrimers. After 96 h, the cells were harvested and counted in the Z2 particle counter (Beckman-Coulter, Indianapolis, IN, USA) to calculate proliferation kinetics. For the estimation of pro-apoptotic potential of the compounds, the cells were seeded at the density of 2 × 10^5^/cm^2^, and treated as described above for 48 h. Subsequently, AnnexinV/Propidium iodide assay was performed (FITC AnnexinV Apoptosis Detection Kit, BD Pharminogen™, San Diego, CA, USA) using ImageStreamX^®^ cytometer (Amnis Corp, Seattle, WA, USA) as described by Ryszawy et al. [42]. Data were analyzed with dedicated IDEAS^®^ 6.2 software (Amnis Corp, Seattle, WA, USA).

### 2.4. Cell Migration Assay

Cells were seeded and treated as described in the proliferation assay section, followed by time-lapse registration starting at 48th h of incubation. Movement of cells were recorded for 8 h with 5 min time interval using a Leica DMI6000B microscope equipped with integrated modulation contrast optics (IMC/integrated modulation contrast, Hoffman contrast, Leica Microsystems, Wetzlar, Germany), CO_2_ chamber (5% CO_2_) and temperature (37 °C) monitoring system. Obtained images were analyzed in Hiro v.1.0.0.4 (written by W. Czapla; Sroka et al., 2007, [43]) by manual cell trajectory tracking (N ≥ 40), followed by calculation of cell motility parameters (speed (µm/min) and displacement (µm)).

### 2.5. Immunocytochemistry

Cells (2 × 10^4^/cm^2^) were cultured in 12-well plates on sterile coverslips and treated with indicated concentrations of dendrons or bola dendrimers for 48 h. Immunolocalization of chosen cellular structures was performed according to previously described protocol [44]. Briefly, cells were fixed with 3.7% formaldehyde (20 min. at room temp.) followed by 0.1% Triton X-100 permeabilization (7 min. at room temp.) and blocking of non-specific binding sites with 2% BSA (30–45 min in 37 °C). Specimens were incubated for 45 min in the presence of monoclonal mouse anti-α-tubulin IgG (T9026, Sigma) primary antibody diluted at volume ratio 1:300 in 2% BSA with 0.01% Tween. After washing with BSA solution, secondary antibody AlexaFluor488-conjugated donkey anti-mouse IgG (Invitrogen; No. A21202, AlexaFluor546-conjugated phalloidin (Invitrogen, No. A22283) for F-actin fibers staining and Hoechst 33258 (Sigma) for DNA staining were applied. Images were acquired with a Leica DMI6000B fluorescence microscope equipped with a DFC360FX CCD camera (Leica Microsystems, Wetzlar, Germany).

## 3. Results

### 3.1. Design and Synthesis

**Figure 1:**
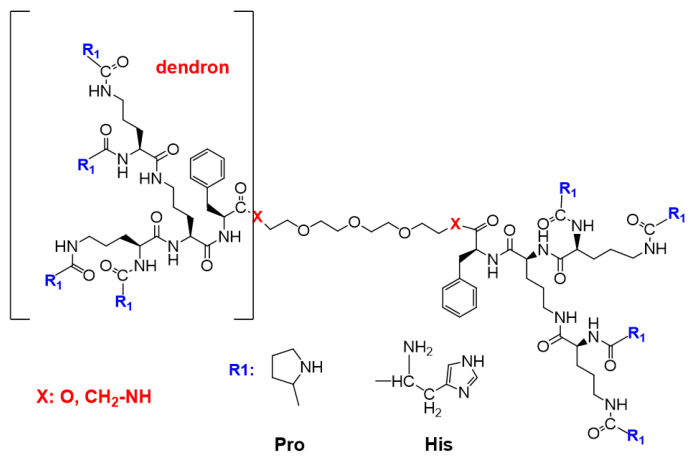
General formula of the obtained dendrimers with bola structure: Orn-based dendrons depicted in brackets were linked via stable amide or biodegradable ester bonds to the linker.

The design strategy was tested against two pathways involving the combination of divergent and convergent methods routinely applied in dendrimer chemistry for obtaining the compounds with a more complex structure [45]. The first strategy was based on the design of fully functionalized N-protected dendrons and coupling them with the linker. The second strategy involved the design of Boc-protected Orn-based dendrons then their dimerization by coupling with the linker, followed by the functionalization of the eight deprotected terminal amino groups with suitably protected histidine (His) or proline (Pro). In both approaches to the dimerization step, the addition of phenylalanine (Phe) at C-terminus of the dendron afforded shorter reaction times and slightly better yields. Finally, the second strategy was applied for obtaining four bola dendrimers with the general formula shown in Figure 1.

A brief overview of the synthesis is shown in the following chapters. A full description of the synthesis along with ^1^H and ^13^C NMR data is available in Supplementary Materials: Synthesis S1 and S2. Monodispersity of the dendrimeric compounds was confirmed by analytical HPLC profiles that are available in Supplementary Materials as Figure S1 (bola dendrimers) and Figure S2 (dendrons).

#### 3.1.1. Synthesis of Ornithine Dendron **4**

**Scheme 1:**
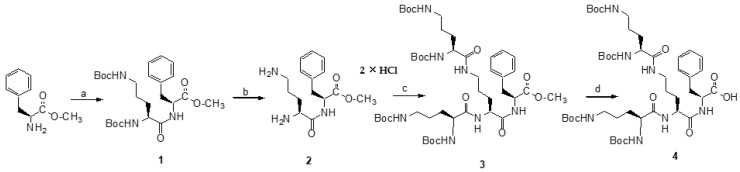
Synthesis of Boc-protected Dendron **4**. Reagents, conditions: (**a**) Boc-Orn(Boc)-OH, DCC/HOSu, THF, 72 h., r.t., yield 90.8%; (**b**) HCl/EtOAc, 4 h, r.t., yield 98.2%; (**c**) Boc-Orn(Boc)-OH, DCC/HOSu, THF, 72 h, r.t., yield: 94.1%; (**d**) 1 M NaOH, 6 h, r.t., yield 89.4%. Note: DCC—*N*,*N*′-Dicyclohexylcarbodiimide; HOSu—*N*-Hydroxysuccinimide.

As shown in Scheme 1, a divergent approach was used to synthesize an ornithine-based Dendron **4** using *N*,*N*′-Dicyclohexylcarbodiimide (DCC) as a coupling reagent with *N*-Hydroxysuccinimide (HOSu). The methyl ester of Boc-Orn(Boc)-Phe with free amino groups **2** was dissolved in THF, then the 10% excess/amine group of the corresponding active ester (i.e., Boc-Orn(Boc)-OSu) in THF was successively added. The reaction was carried out at room temperature for 3–5 days and monitored on TLC plates. Afterward, the solvent was evaporated to dryness and the post-reaction mixture was dissolved in ethyl acetate and washed several times with 5% citric acid solution, saturated aqueous NaHCO_3_ and brine, dried over MgSO_4_, filtered and evaporated in vacuo. The compounds were purified on Silica Gel or on Sephadex LH-20 and preparative high performance liquid chromatography (HPLC), then the solvent was removed in vacuo to give the desired compound **4** as a light yellow oil. The structure was confirmed on the basis of ^1^H and ^13^C NMR spectra.

#### 3.1.2. Synthesis of Dendrimer Intermediates with Bola Structures **7** and **8**

**Scheme 2:**
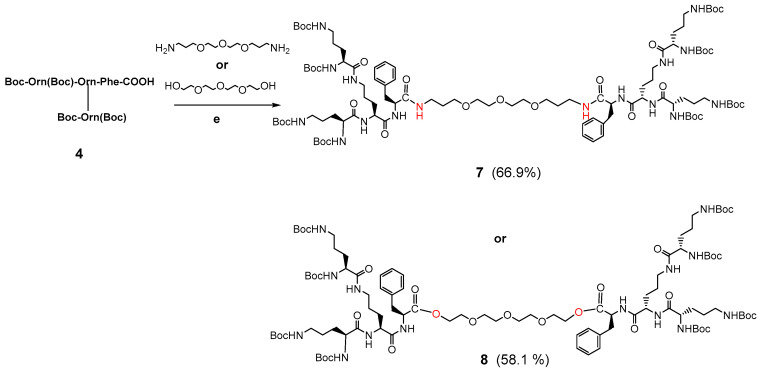
Synthesis of Boc-protected bola dendrimer **7** connected with amide bonds or Boc-protected bola dendrimer **8** connected with ester bonds. Reagents, conditions: (**e**) DCC/HOSu, MeOH/THF, 96 h, r.t., 66.9% yield for **7** or DCC/DMAP/THF, 96 h, r.t., 58.1% yield for **8**.

Synthesis of the Boc-protected dendrimer **7** and **8** was performed by coupling 1.1 equivalent of the respective Dendron **4** in an active ester form with 0.5 equivalent of 4,7,10-trioxa-1,13-tridecanediamine in THF to obtain crude **7** (for reagents and conditions see Scheme 2). Synthesis of the bola intermediate **8** with biodegradable ester linker involved coupling 1.1 equivalent of the respective Dendron **4** with 0.5 equivalent of tetraethylene glycol dissolved in THF. The raw dendrimeric compounds were purified by molecular filtration on the Sephadex LH-20 packing with MeOH as eluent and then by preparative HPLC.

#### 3.1.3. Synthesis of Bola Dendrimers **9a** and **10a**

**Scheme 3:**
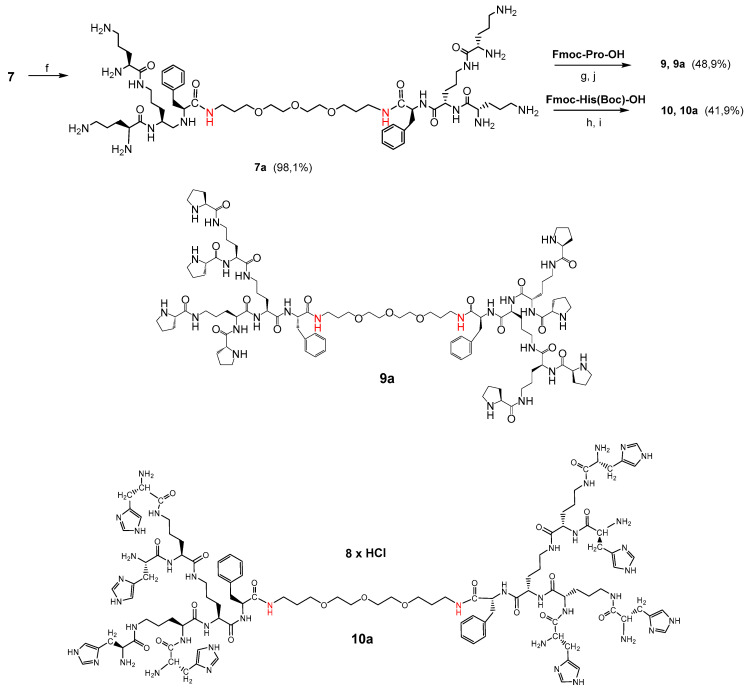
Synthesis of bola dendrimers **9a** and **10a**. Reagents/conditions: (**f**) HCl/EtOAc, 8 h, r.t.; (**g**) Fmoc-Pro-OH, DCC/HOSu, THF, 126 h, r.t.; (**h**) Fmoc-His(Boc)-OH, DCC/HOSu, THF, 126 h., r.t.; (**i**) HCl/EtOAc, 8 h, r.t.; (**j**) tris(2-aminoethyl)amine/MeOH, 8 h, r.t., yield 48.9% for **9a** and 41.9% for **10a**. Note: Pro—proline; His—histidine.

**Scheme 4:**
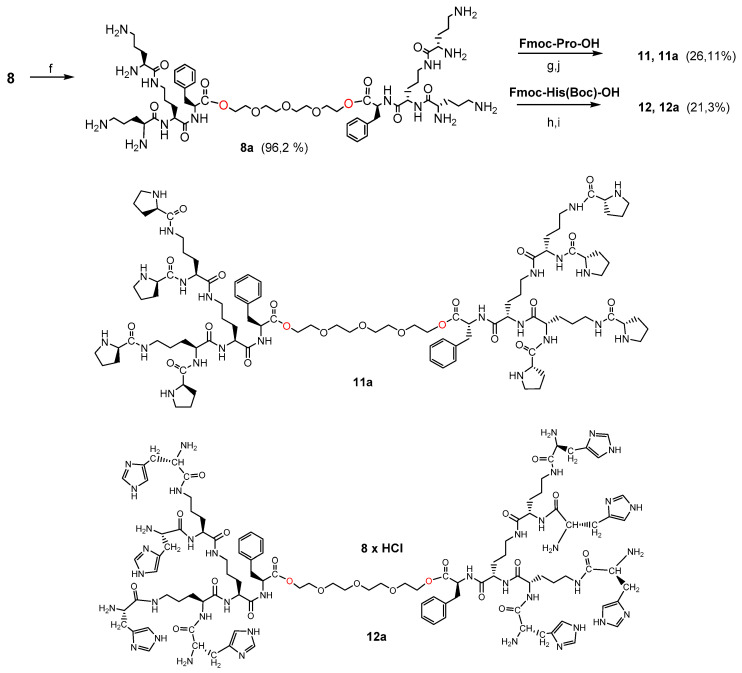
Synthesis of bola dendrimers **11a** and **12a**. Reagents/conditions: (**f**) HCl/EtOAc, 8 h, r.t.; (**g**) Fmoc-Pro-OH, DCC/HOSu, THF, 126 h, r.t.; (**h**) Fmoc-His(Boc)-OH, DCC/HOSu, THF, 126 h, r.t.; (**i**) HCl/EtOAc, 8 h, r.t.; (**j**) tris(2-aminoethyl)amine/MeOH, 8 h, r.t.

In the first step, the dendrimers **7** or **8** were converted to octahydrochlorides by Boc-deprotection with HCl-saturated AcOEt. The final bola dendrimers terminated with proline groups **9** and **9a** were obtained by coupling 8.8 equivalent of Fmoc-Pro-OH with the respective bola intermediates **7a** or **8a** (for reagents and conditions see Scheme 3). For the final bola dendrimers **10** and **10a** terminated with histidine 8.8 equivalent of Fmoc-His(Boc)-OH in the form of active ester were coupled with the respective bola intermediates **7a** or **8a**. After purification by size exclusion chromatography on Sephadex LH-20 followed by preparative HPLC, the dedrimers were converted to octahydrochlorides by removing of the Boc groups with HCl-saturated AcOEt (Scheme 4).

#### 3.1.4. Synthesis of Monomeric Dendrons **13**, **14** and **15**

**Scheme 5:**
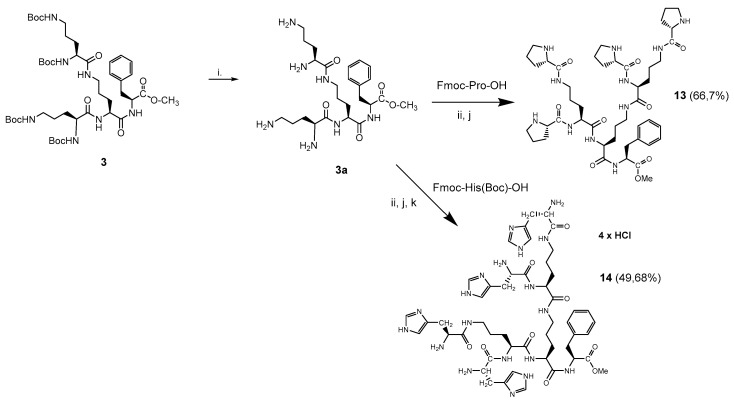
Synthesis of peptide dendrons **13** and **14** in solution: (**i**) HCl/EtOAc, 8 h, r.t.; (**ii**) Fmoc-Pro-OH, DCC/HOSu, THF, 126 h, r.t.; or Fmoc-His(Boc)-OH, DCC/HOSu, THF, 126 h, r.t.; (**j**) 20% piperidine/MeOH; (**k**) HCl/EtOAc, 8 h, r.t.

**Scheme 6:**
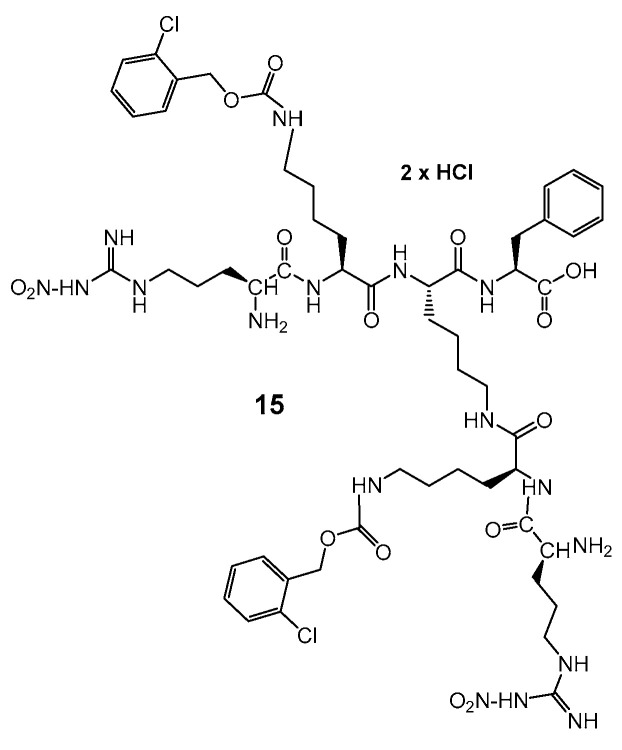
Structure of lysine Dendron **15,** functionalized with Arg-NO_2_.

In order to compare the impact of bola type dimerization on anti-glioblastoma activity, two monomeric dendrons **13, 14** were synthesized by the application of typical Boc-chemistry in solution (Scheme 5). Dendron **15** (Scheme 6) was synthesized on solid support according to the procedure described in Supplementary Material: Synthesis S2. Monomeric derivatives were designed to test their capability to interact or possibly to cross membranes of the model glioblastoma cells. Even though dendron **13** with Pro-terminated arms is neutral, AMP’s with four prolines have the ability to cross the blood–brain barrier (BBB) [41,42]. The positively charged and amphiphilic dendrons **14** and **15** contained either four histidine or two nitro-arginine residues, that might provide a cell penetrating function. Moreover, arginine constitutes the tumor targeting RGD sequence.

### 3.2. Circular Dichroism (CD) Studies on Molecular Conformation

Primary idea for the design of amphiphilic bola compounds was emulation due to self-assembly of the higher order structures with tunable morphology (fibers, rods, tubes) and generation of desired physical properties [46]. With this regard, structural studies utilizing circular dichroism (CD) methods for compounds containing formal stereogenic centers (e.g., connected by hydrophobic linkers l-*α*-aspartyl-l-phenylalanine or valyl-valine heads) [47,48] were applied to trace the transfer of molecular chirality to the secondary structures.

**Figure 2:**
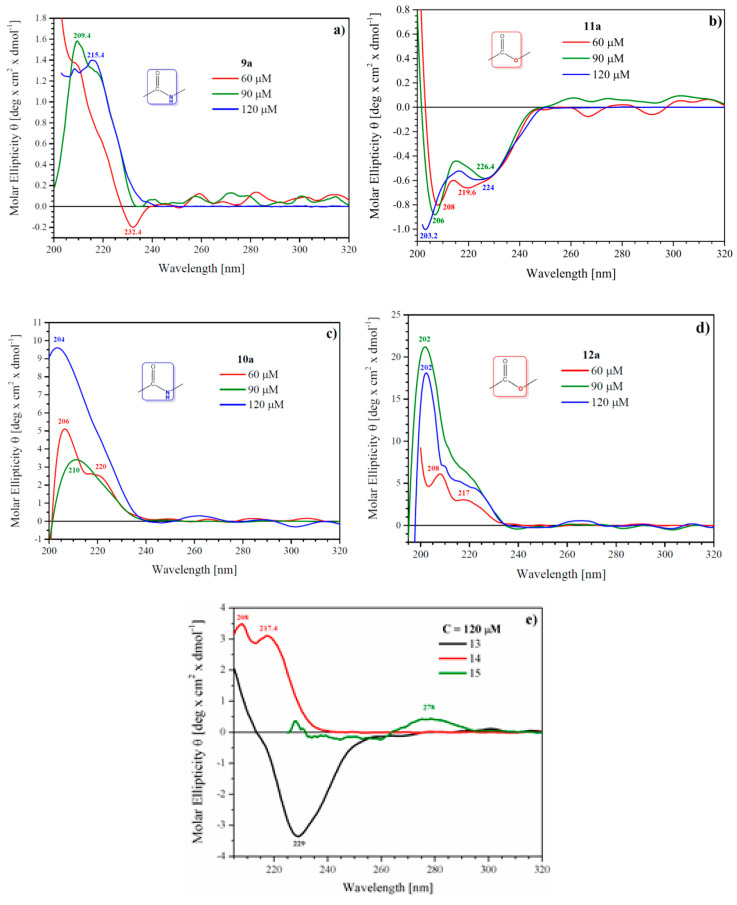
Circular dichroism spectra of proline-decorated bola dendrimers **9a** and **11a** (**a**,**b**), histidine-decorated bola dendrimers **10a** and **12a** (**c**,**d**) and monomeric dendrons **13**–**15** (**e**) showing concentration and structure dependent circular dichroism (CD) trace.

As molecular self-assembly of the bola-type dendrimers can be significant for biological activity and for the prospective carrier properties, CD spectroscopy was used to probe concentration-dependent supramolecular aggregation in solution. Circular dichroism spectra for Pro-decorated **9a** and **11a** and His-decorated bola dendrimers **10a** and **12a** show patterns that are characteristic also for the respective monomeric dendrons: Pro-decorated **13** or His-decorated **14** (Figure 2). CD spectra measured in MeOH revealed that none of the bola dendrimers except Pro-decorated **11a** show CD trace characteristics for linear AMP’s, where α-helix conformation allows sorting of cationic and hydrophobic centers. With this respect, the amphipathic structure of cationic His-terminated bola’s **10a** and **12a** is due to chemical design rather than conformational rearrangement(s). All spectra show positive Cotton effect of the highest intensity around 190–206 nm, more intense for His-decorated dendrimers, characteristic for n-π* transition of the amide bond in various chemical environments. From the two neutral Pro-decorated bola’s, only **11a** shows CD trace with negative bands around 208, 219 and a saddle at 226 nm. This may account for a mixture of an α-helix and β-sheet secondary structures in MeOH solution. The β-sheet structure might also be postulated for monomeric Dendron **13**, showing a broad negative band at 239 nm. A quite different shape of the CD spectrum for dendrimer **9a** that structurally differs from **11a** by just two ester bonds at a linker—dendron connection suggests that intermolecular interactions are driven by non-covalent head-to-head and head-to-linker interactions. His-decorated dendrimers **10a** and **12a** as well as monomeric dendron **13** show positive bands around 206 and 220 nm at 60 μM concentration. At higher concentrations, they lose discrete structure and are replaced by a broad positive band with maximum at 202–204 nm that probably covers a range of secondary structures developed during aggregation of molecules with a slightly different location of histidine side chains.

In summary, the CD spectra suggest that the bola dendrimers exist in MeOH solution as a mixture of aggregates with multiple conformations. In the case of His-rich dendrimers, multiple hydrogen bonds between imidazole fragments probably mediated by Cl anions can be postulated. CD spectra of all dendrimers also contain minima around 200 nm, which might suggest the presence of aggregates with random conformation, at least for Pro-rich bola dendrimers. As the CD spectrum of L-His contains a sharp minimum at 195.8 nm, random conformation of His-rich derivatives is less reliable.

### 3.3. Activity of Pro-Functionalized Dendrons and Their Dimeric Bola Dendrimers in Glioblastoma In Vitro Model

**Figure 3:**
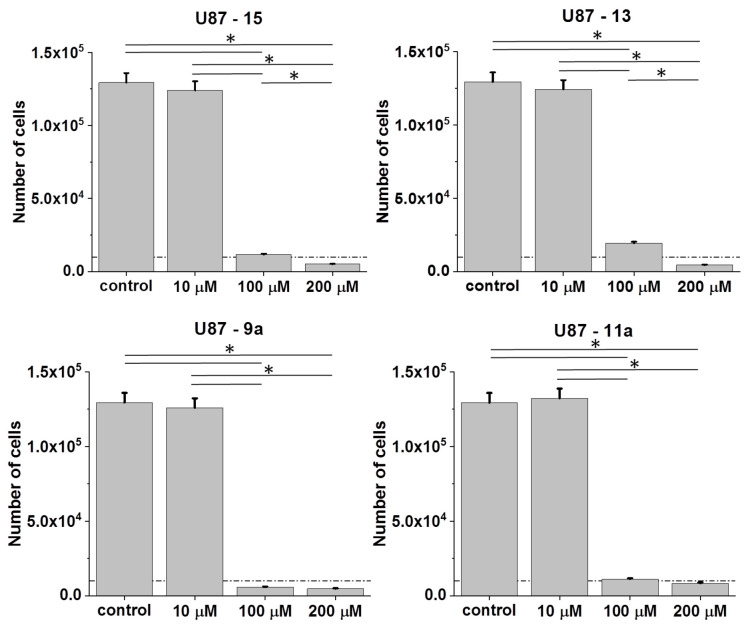
Cytostatic effects of cationic **15** and neutral Pro-functionalized monomeric dendron **13** and Pro-functionalized bola dendrimers **9a** and **11a** in U87 populations. Cells were cultured for 96 h after the administration of the agents (1–200 μM) and their numbers were counted with Coulter counter. All data shown as mean ± SD. Statistical significance of the differences tested with two-way ANOVA, * *p* < 0.01. Data representative for at least 3 independent experiments (*n* > 3). Note the relatively high bioactivity of Pro-functionalized compounds, and the relatively high sensitivity of U87 cells to the analyzed agents.

Cytostatic and the pro-apoptotic activity of ornithine dendrons/dendrimers determine their anti-tumorigenic potential. To estimate interrelations between their structure and bioactivity two dendrons: cationic, Arg-NO_2_-terminated Dendron **15** and Pro-functionalized Dendron **13** were tested in a U87 glioblastoma cell model (Figure 3). A relatively strong inhibitory effect of (+)4 charged Arg-NO_2_-terminated dendron **15** on U87 cell proliferation was detected, even though its activity was seen only at the concentrations of 100 and 200 μM.

Pro-substitution and the resulting neutral character of the dendron **13** attenuated its cytostatic activity, as illustrated by the comparison of cell numbers in the presence of 100 μM of **15** and **13**, respectively. Interestingly, Pro-functionalized bola dendrimer **9a** connected with the linker via two amide bonds displayed higher cytostatic activity than both monomers, when administered at the concentration of 100 μM. However, binding the same dendron by two cleavable ester bonds as in the bola dimer **11a** lowered its activity. These data suggest that dendron dimerization can increase its bioactivity, whereas both molecular charge and particular chemical structure (amide/ester bonds at linker connection) modulate this effect.

**Figure 4:**
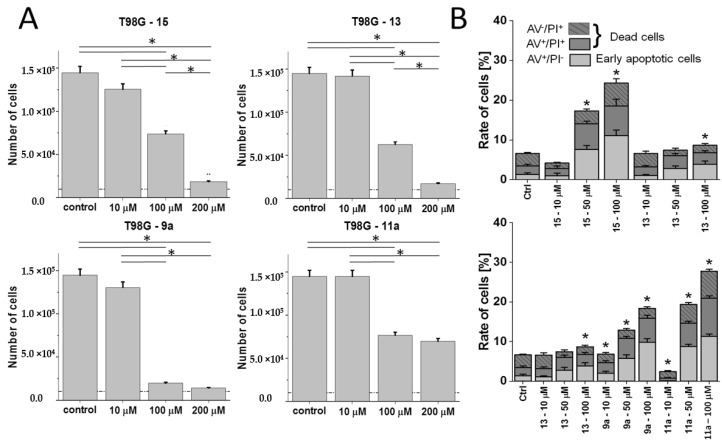
Cytostatic and anti-apoptotic effects of cationic 15 and neutral Pro-functionalized monomeric dendron **13** and bola dendrimers **9a** and **11a** in T98G populations. (**A**) Cells were cultured for 96 h after the administration of the agents (1–200 μM) and their numbers were counted with a Coulter counter. (**B**) T98G cells were treated with **15**, **13**, **9a** and **11a** (1–100 μM) and the fraction of apoptotic cells was estimated with ImageStream-AnnexinV/PI assay after 48 h based on the compensated dot-plots, which comprised 50,000 events (Figure S3 in the Supplementary Materials). All data shown as mean ± SD. Statistical significance of the differences tested with Two-way ANOVA, * *p* < 0.01. Data representative for at least 3 independent experiments (*n* > 3). Note the lower sensitivity of T98G cells to the analyzed agents in comparison to their U87 counterparts and the lack of straightforward correlation between cytostatic and pro-apoptotic activity of the compounds.

To verify this notion, we further compared the activity (cytostatic and pro-apoptotic propensity) of dendrons/dendrimers in an alternative T98G cellular model, which is characterized by higher intrinsic heterogeneity. This is illustrated by the co-existence of discrete sub-populations that differ in morphology, invasive potential and, conceivably, drug resistance (D.R.; unpublished data). Again, both dendrons (**15** and **13**) as well as bola dendrimers **9a** and **11a** displayed a concentration-dependent cytotoxicity pattern; even though T98G cells were less vulnerable to their action than U87 cells (Figure 4A). Dimer **11a** was most sensitive to phenotypic differences between these two cell lines as illustrated by relatively high T98G numbers even at the 200 μM concentration.

Real time FITC-Annexin V/ethidium bromide assay was used for determination of the cell death characteristics. Here, we introduced 50 µM concentration to more accurately identify the concentrations of the compounds that result in the significant apoptotic response of the cells. As shown in Figure 4B, dendron **15** exerted a more pronounced apoptotic effect than **13** in the T98G model in the absence of significant differences in their overall cytostatic activity (Figure 4B; cf. Figure S3 in Supplementary Materials). Dimerization of the Pro-decorated dendron **13** into the bola dimer **9a** increased both the pro-apoptotic and cytostatic activity of the compounds. Interestingly, the introduction of an ester instead of amide moieties at the dendron–linker connection (**11a**) lowered the cytostatic effect, but not its pro-apoptotic activity. These data can be explained in terms of the heterogeneity of T98G populations that may underlie their heterogonous sensitivity to the compounds. Strong apoptotic responses of super-sensitive T98G sub-population(s) can be compensated by the lower sensitivity of other T98G sub-population(s) to the considered compound.

### 3.4. Effect of Pro-Functionalized Dendrons/Dendrimers on the Invasive Potential and Morphology of T98G Cells

Low sensitivity of tumor cells to chemotherapy often correlates with their increased invasiveness [49]. To determine how the chemical structure of dendrons affects their impact on the invasiveness of phenotypically heterogeneous glioblastoma cells, we estimated the morphology and motility of the T98G cells treated by **15** and **13**.

**Figure 5:**
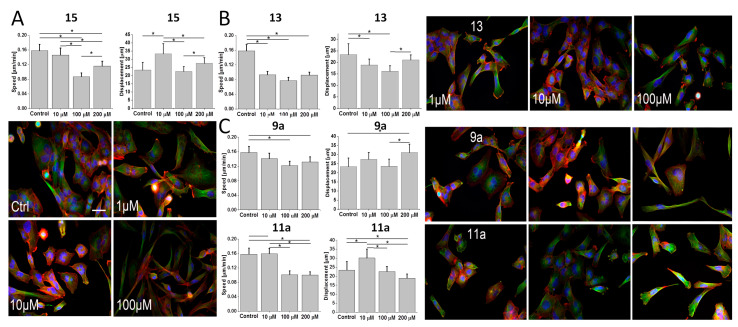
Effect of Pro-functionalized dendrons/dendrimers on the invasive potential and morphology of T98G cells. (**A**) T98G cells were treated with **15** (1–200 μM) and their motility was estimated with time-lapse videomicroscopy after 48 h to construct single cell trajectories from the sequences of cell centroid positions (see Materials and Methods; cf. Figure S4 in the Supplementary Materials). Parameters of trajectories were further compiled to calculate the averaged values of speed of the cell movement (Speed: distance/recording time; μm/h) and total length of single cell displacement (Displacement; μm; from >3 independent experiments; number of cells > 50). Concomitantly, the cells were stained against F-actin (red) and alpha-tubulin (green), and counterstained against DNA (Hoechst 33258) to visualize the effect of **15** on cell morphology. Scale bar: 50 μm. (**B**,**C**) Cells were cultivated in the presence of **13** (**B**) or in the presence of **9a** and **11a** (**C**) and processed as in A. All data shown as mean ± SD. Statistical significance of the differences tested with two-way ANOVA, * *p* < 0.01. Data representative for at least 3 independent experiments (*n* > 3). Note the spindle-like shape of T98G cells in the presence of higher 15 and 9a concentrations, which is accompanied by the induction of their displacement.

A relatively negligible inhibitory effect of **15** on T98G motility was accompanied by its strong effect on the morphology of T98G cells, as illustrated by their shift towards spindle-like morphology in the presence of 100 μM **15** (Figure 5A). Less pronounced phenotypic shifts and relatively negligible inhibition of T98G motility were observed in the presence of **13** (Figure 5B). Interestingly, effects of Pro-functionalized bola dendrimers **9a** and **11a** on T98G motility (Figure 5C) hardly correlated with their cytostatic/pro-apoptotic activity (cf. Figure 4). Negligible effects of **11a** on T98G proliferation and morphology were accompanied by generally lower T98G motility under 100 μM **11a** stress. In turn, relatively high cytostatic activity of **9a** correlated with increased displacement rates of **9a**-treated T98G cells and their spindle-like/rear-front polarized morphology under 100 μM **9a** stress (Figure 5C). These data confirm that Pro-functionalized, but structurally different dendrons/dendrimers prompt the discrete pattern of phenotypic microevolution/selective expansion of drug-resistant/invasive cells in heterogeneous glioblastoma populations. The opposite effects of heterogeneous T98G chemoresistance and dendron/dendrimer cytotoxicity may add to less pronounced bioactivity of dendrons/dendrimers in the T98G system.

### 3.5. Impact of His-Functionalized Amphiphilic Bola Dendrimers on the Cytostatic Activity

**Figure 6:**
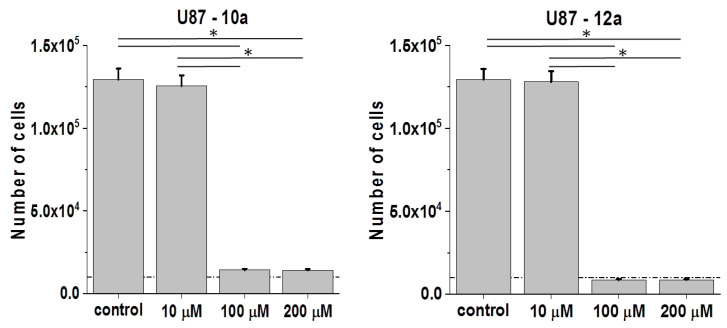
Effect of His-functionalized bola dendrimers on the bioactivity of U87 glioblastoma cells. U87 cells were cultivated in the presence of **10a** or **12a** (1P200 μM) for 96 h after the administration of the agents and their numbers were counted with a Coulter counter. All data are shown as mean ± SD. Statistical significance of the differences tested with Two-way ANOVA, * *p* < 0.01. Data representative for at least 3 independent experiments (*n* > 3). Scale bar: 50 μm. Note the relatively high cytostatic activity of ester-bound amphiphilic dendrimer 12a in the U87 model.

Contrary to the non-charged, Pro-terminated bola dimers **9a** and **11a**, His-functionalized bola analogs **10a** and **12a** are (+8)-charged and amphiphilic. Studies on **10a** (His-substituted) bola dendrimer in U87 glioblastoma model revealed its cytostatic activity, which was similar to that of its amide bond linked **9a** (Pro-substituted) analog (Figure 6; cf. Figure 3). In the second pair, where dendrons are connected via ester bonds, we saw a slightly higher sensitivity of U87 cells to **12a** than to **11a.** Notably, the activity of **12a** was also higher than that of **10a**, which indicates the importance of the combination of ester bonds/amphiphilic properties for dendron/dendrimer activity.

**Figure 7:**
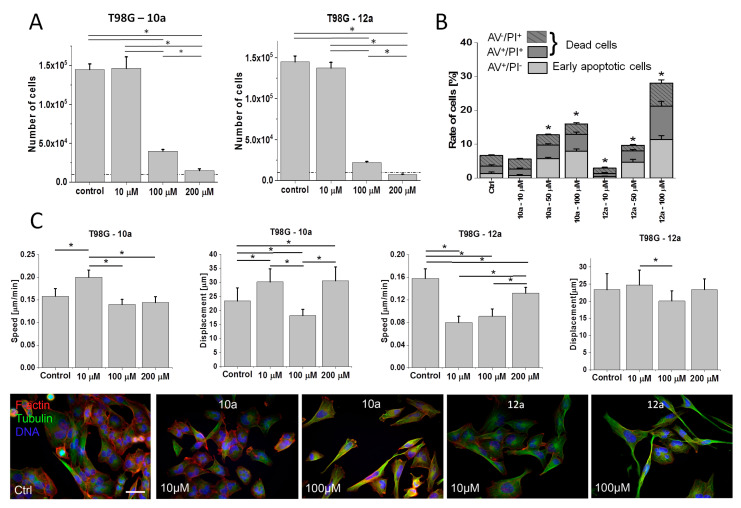
Effect of His-functionalized bola dendrimers on the bioactivity of T98G glioblastoma cells. (**A**) T98G cells were cultivated in the presence of **10a** or **12a** (1–200 μM) for 96 h after administration of the agents and their numbers were counted with a Coulter counter. (**B**) T98G cells were treated with **10a** or **12a** (1–100 μM) and the fraction of apoptotic cells was estimated with ImageStream-AnnexinV/PI assay after 48 h (Figure S3 in Supplementary Materials). (**C**) (Upper panel) T98G cells were treated with **10a** and **12a** (1–200 μM) and their motility was estimated with time-lapse videomicroscopy after 48 h of treatment (Figure S4 in Supplementary Materials). (Lower panel) Comparison of the morphology and actin (green)/microtubular cytoskeleton (red) architecture of T98G cells cultivated in control conditions and under **10a/12a** stress. All data shown as mean ± SD. Statistical significance of the differences tested with two-way ANOVA, * *p* < 0.01. Data representative for at least 3 independent experiments (*n* > 3). Scale bar: 50 μm. Note the relatively high cytostatic activity of **12a** in the T98G model.

Relatively high bioactivity of **12a** was also observed in the more resistant T98G model, as illustrated by the comparison of T98G proliferation in the presence of **10a** and **12a** (Figure 7A). It was accompanied by a higher pro-apoptotic activity of 100 μM **12a** (Figure 7B; cf. Figure S3 in Supplementary Materials), and the lack of the dose-dependence of **10a**/**12a** effects on T98G motility (Figure 7C, cf. Figure S4 in Supplementary Materials). In the pair of ester-bound dendrimers (**11a** and **12a**), histidine residues apparently enhanced anti-proliferative and anti-invasive activity of **12a** in T98G model (Figure 7A,C; cf. Figure 4A,C), even though Pro-substituted analog **11a** displayed similar pro-apoptotic activity to its His-decorated **12a** derivative (Figure 7B; cf. Figure 4B). Reduced proliferation of T98G cells in the presence of **12a** was accompanied by their reduced motility (speed) in the presence of 10 μM **12a**. Notably, contrary to **11a**, His-decorated **12a** increased the speed of T98G cells and the fraction of spindle-like T98G cells when applied at 200 μM (in comparison to 10 μM; Figure 7C). It confirms that a fraction of highly motile T98G cells can display increased resistance to both agents.

On the other hand, two bola dimers of different chemical structure and periphery, but of similar cytostatic activity: Pro-decorated **9a** (Figure 4) and His-decorated **12a** (Figure 7) evoked increased motile activity of T98G cells, accompanied by increased fraction of spindle-like cells. Collectively, our data show that His-substitution (amphipathic structure/positive charge) can increase the bioactivities of dendrimers; however, this effect can be counteracted by cellular heterogeneity and microevolution of drug-resistant glioblastoma populations.

### 3.6. Discussion

A series of bioinspired monomeric ornithine dendrons and dimeric dendrimers with bola structure was designed and tested for their effects on the proliferation and invasiveness displayed by two phenotypic glioblastoma cell lines U87 and T98G. From the chemical and biomedical perspective, dendrimers with bola-type structure have several advantages over much larger dendrimers of high generations. Chemically, such molecules can be easier to synthesize and purify, providing monodisperse material valuable for biomedical applications. Bola dimerization gives the opportunity to modulate the desired properties by combination of two identical or two structurally different fragments and to obtain higher molecular mass molecules in a controlled reaction pathway resulting in their longer retention in body fluids. Proline-functionalized dendron and dendrimers of the bola-structure are not charged molecules and, therefore, they are less likely to express typical cationic antimicrobial and anticancer peptide interactions with the negatively-charged membrane components [12]. In turn, carrying eight positively-charged amphipathic bola dendrimers functionalized with histidine might interact with cancer cell membranes. Although the mechanism by which they affect cancer cells is not completely understood, it is probable that they discriminate among the cells based on the electrostatic interaction between the charged amino acids of the dendrimer and the negatively-charged membrane components present in excess in cancer cells [50].

**Figure 8:**
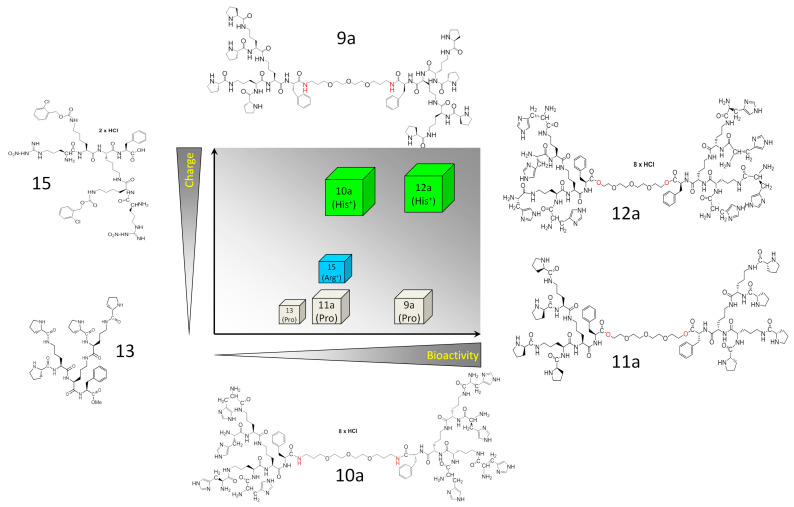
Schematic diagram the summarizing the interrelations between the mass, charge and bioactivity of the studied compounds.

Our current data confirm the role of dimerization/charge of the ornithine dendrons in the determination of their bioactivity in glioblastoma systems (Figure 8). However, they also show the complexity of cell reactions and the role of cellular context/microevolution in glioblastoma cell reactivity to these compounds.

Intracellular bioavailability of dendrimeric compounds, which primarily determines their anti-tumor activity, depends on their ability to penetrate cellular membranes. This is positively correlated with the (positive) charge of dendrimers and often depends on their size (molecular mass). In general, small, positively charged biomolecules more easily penetrate biomembranes than their neutral counterparts [51]. Our data are partly in agreement with this notion, because positively charged NO_2_-arginylated ornithine dendron **15** exerted more pronounced cytostatic effects on U87 than its Pro-functionalized (neutral) counterpart **13**. On the other hand, we observed a significant cytostatic activity of Pro-functionalized monomeric **13** and bola dendrimers functionalized with proline (**9a** and **11a**). Even though Pro-substitution of dendrimers interferes with this effect, our data indicate that neutral charge does not erase their activity. Probably, Pro-functionalized compounds can also be actively internalized by the glioblastoma cells. For instance, proline residue(s) can affect the efficiency of endocytotic pathways, modifying intracellular fates of dendrons/dendrimers and their targeting, as described for other peptides [52].

Previously, bioavailability of dendrimers has been negatively correlated with their size (molecular mass) [53]. Apparently, this parameter influences their ability to penetrate biological barriers (including BBB) and to interfere with intracellular pathways. However, dendrons dimerization into bola dendrimers yielded more active derivatives. This is illustrated by the higher activity of **9a** vs. **13** in both cellular systems. Multiple positive charge and apparent amphipathic character is also important since replacement of proline with histidine significantly increased the bioactivity of the dendrimers. This is illustrated by the relatively high cytostatic activity of **12a** in comparison to **11a** in both T98G and U87 cell lines. Structural modification concerning linkage of the monomeric dendrons by the chemically more stable amide (**9a** and **10a**) or potentially cleavable ester bonds (**11a** and **12a**) is less clear and is probably associated with the type of terminal amino acid. In two pairs of Pro/His-substituted compounds, the pair of dimers connected with an ester bond is two times less (Pro) or twice as active (His) as the appropriate amides. These observations confirm that the positive charge and amphipathic character of ornithine dendrons/dendrimers plays a primary role in the regulation of their activity.

Collectively, the size and charge of dendrons/dendrimers potentially affects their bioavailability and the efficiency of direct interactions with intra- and intercellular signaling systems that accounts for their cytostatic activity (Figure 8). However, different sensitivity of U87 and T98G cells to the presence of ornithine dendrons/dendrimers suggests that their bioactivity also depends on “cellular context”. In particular, discrepancies between pro-apoptotic and cytostatic activity of **15** and **13** indicate the heterogeneous response of T98G to these compounds. Apparently, sub-population(s) of hyper-sensitive cells co-exist(s) with drug-resistant T98G sub-population(s). Consequently, strong apoptotic responses of one T98G sub-population can be compensated by the lower sensitivity of other T98G sub-population(s) to the considered compound. The presence of hyper-resistant T98G sub-population(s) is indicated by a correlation between the inhibition of T98G cell proliferation and induction of their motility in the presence of **9a** and **12a**. Although further studies are necessary to comprehensively scrutinize these interrelations, **9a**/**12a**-induced selection of the “post-EMT” cells may account for the shifts in cell morphology. Epithelial-mesenchymal transition (EMT) results in increased motility/invasiveness of cancer cells and often increases their drug-resistance [49]. Even though glioblastoma is not an “epithelial” tumor, the corresponding process of “Glial-MT” may account for the decreased T98G cell reactivity to the compounds, thus facilitating their clonal expansion in stress conditions. This could also provide the explanation for the abundance of rear-front polarized cells in **9a**/**12a**-treated T98G populations. In turn, growth inhibition and spindle-like morphology of **15**/**13** and **12a**-treated T98G cells indicates that these compounds can impair the adhesion of epithelioid glioblastoma cells, thus inhibiting their proliferation. Collectively, our data confirm that the size, charge and amphipathic character of ornithine dendrons/dendrimers play a primary role in the regulation of their activity. They also identify these compounds as a group of promising anti-tumor agents that can serve as tools to study interrelations between drug bioactivity, its chemical properties and tumor cells’ phenotype.

## Supplementary Materials

The following are available online at https://www.mdpi.com/1999-4923/12/11/1106/s1, Figure S1: Analytical HPLC profiles for bola dendrimers **9a**, **10a**, **11a**, **12a**; Figure S2: Analytical HPLC profiles for dendrons **13**–**15**; Figure S3: pro-apoptotic effects of ornithine dendrons/dendrimeres in T98G cell populations. Cells were seeded at the density of 2 × 10^5^/cm^2^. After 24 h of initial incubation, the tested agents were applied at the concentration of 100 μM in the fresh portion of medium for the next 48 h. Subsequently, AnnexinV/Propidium iodide assay was performed (FITC AnnexinV Apoptosis Detection Kit, BD Pharminogen™, San Diego, CA, USA) using ImageStreamX^®^ cytometer (Merck Millipore, Burlington, MA, USA). Data representative for at least 3 independent experiments (*n* > 3) were analyzed with IDEAS^®^ 6.2 software (Merck Millipore); Figure S4: Exemplary plots showing the effect of ornithine dendrons/dendrimers on the motility of T98G cells. Cells were seeded at the density of 2 × 10^4^/cm^2^. After 24 h of initial incubation, the tested agents were applied at the concentration of 10–100 μM and cell movement was registered 48 h afterwards with time-lapse videomicroscopy. Cell trajectories are depicted in circular diagrams (axis scale in µm) drawn with the initial point of each trajectory placed at the origin of the plot (registered for 6 h; *n* > 50). Data representative for at least 3 independent experiments (*n* > 3); Synthesis and NMR data: Synthesis S1 and Synthesis S2; Scheme S1. Synthesis of Boc-protected dendron 4. Reagents, conditions (a) Boc-Orn(Boc)-OH, DCC/HOSu,THF, 72 h., r.t., yield 90.8%; (b) HCl/EtOAc, 4 h, r.t., yield 98.2%; (c) Boc-Orn(Boc)-OH, DCC/HOSu,THF, 72 h, r.t., yield: 94.1% ; (d) 1M NaOH, 6 h, r.t., yield 89.4%; Scheme S2. Synthesis of Boc-protected peptidic bola-dimer **7** connected with amide bonds or Boc-protected bola-dimer **8** connected with ester bonds. Reagents, conditions: (e) DCC/HOSu, MeOH/THF, 96 h, r.t. 66.9%, yield for **7** or DCC/DMAP/THF, 96 h, r.t., yield for 8 58.1%; Scheme S3. Synthesis of functionalized bola-dendrimers **9a** and **10a**. Reagents, conditions: (f) HCl/EtOAc, 8 h, r.t.; (g) Fmoc-Pro-OH, DCC/HOSu,THF, 126 h, r.t.; (h) Fmoc-His(Boc)-OH, DCC/HOSu,THF, 126 h., r.t.; (i) HCl/EtOAc, 8 h, r.t.; (j) 20% piperidine/MeOH 4 h, r.t., yield 48.9% for **9a** and 41.9% for **10a**; Scheme S4. Synthesis of bola-dendrimers 11a and 12a. Reagents, conditions: (f) HCl/EtOAc, 8 h, r.t.; (g) Fmoc-Pro-OH, DCC/HOSu,THF, 126 h, r.t.; (h) Fmoc-His(Boc)-OH, DCC/HOSu,THF, 126 h, r.t.; (i) HCl/EtOAc, 8 h, r.t.; (j) 20% piperidine/MeOH, 4 h, r.t.; Scheme S5. Synthesis of peptide dendrons 13 and 14 in solution: (i) HCl/EtOAc, 8 h, r.t.; (ii) Fmoc-Pro-OH, DCC/HOSu,THF, 126 h, r. t.; or Fmoc-His(Boc)-OH, DCC/HOSu,THF, 126 h, r.t.; (j) 20% piperidine/MeOH; (k) HCl/EtOAc 8 h, r.t.; Scheme S6. Synthesis of peptide dendron 15 on solid suport: (a) 20% piperidine/DMF, (b) Fmoc-Lys(Fmoc)-OH, HATU, DIPEA, (c) 20% piperidine/DMF, (d) Fmoc-Lys(2-Cl-Z)-OH, HATU, DIPEA (e) 20% piperidine/DMF, (f) Fmoc-Arg(NO2)-OH, HATU, DIPEA (g) 20% piperidine/DMF (h) 90% TFA/H2O. Table S1. Chemical data.
