# Supplementary material for: Bioinspired Bola-Type Peptide Dendrimers Inhibit Proliferation and Invasiveness of Glioblastoma Cells in a Manner Dependent on Their Structure and Amphipathic Properties
Source: Pharmaceutics. 2020 Nov 18;12(11):1106. doi: 10.3390/pharmaceutics12111106 (PMC7698760; doi:10.3390/pharmaceutics12111106)
Supplement: Supplementary file 1 [file pharmaceutics-12-01106-s001.pdf]

pharmaceutics

MDPI

Supplementary Materials: Bioinspired Bola-Type Peptide Dendrimers Inhibit Proliferation and Invasiveness of Glioblastoma Multiforme Cells in a Manner Dependent on Their Structure, Amphipathic Properties and Cell Phenotype

Maciej Cieślak [1], Damian Ryszawy [2,†], Maciej Pudełek [2], Magdalena Urbanowicz [1], Maja Morawiak [1], Olga Staszewska-Krajewska [1], Jarosław Czyż [2*] and Zofia Urbanczyk-Lipkowska [1*]

Compound 9a

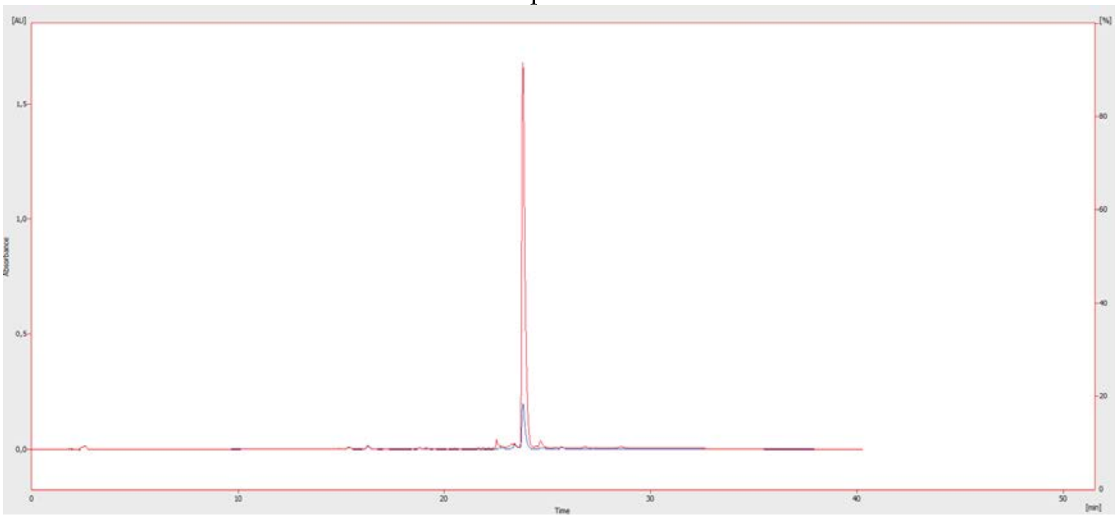

Compound 10a

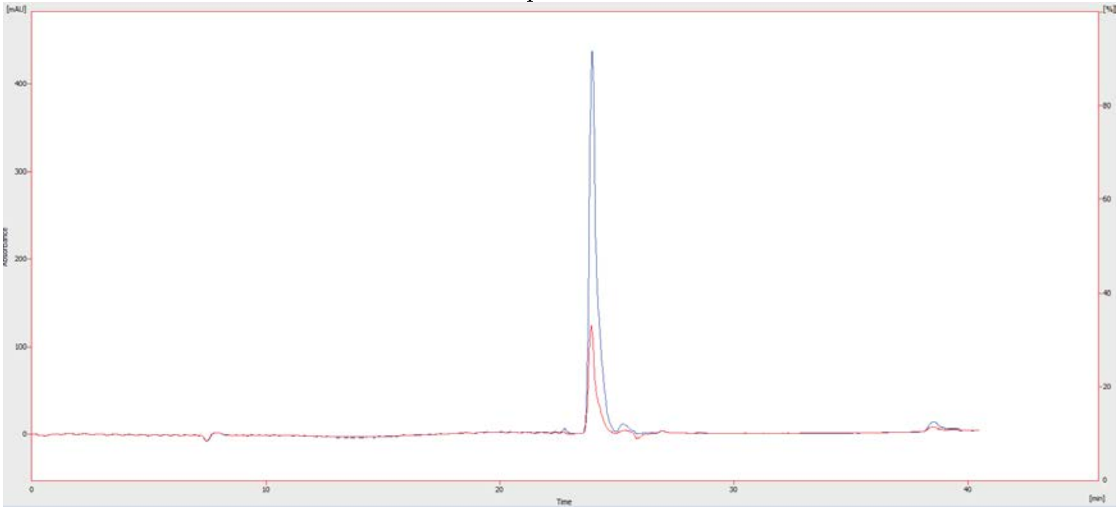

pharmaceutics

MDPI

Compound 11a

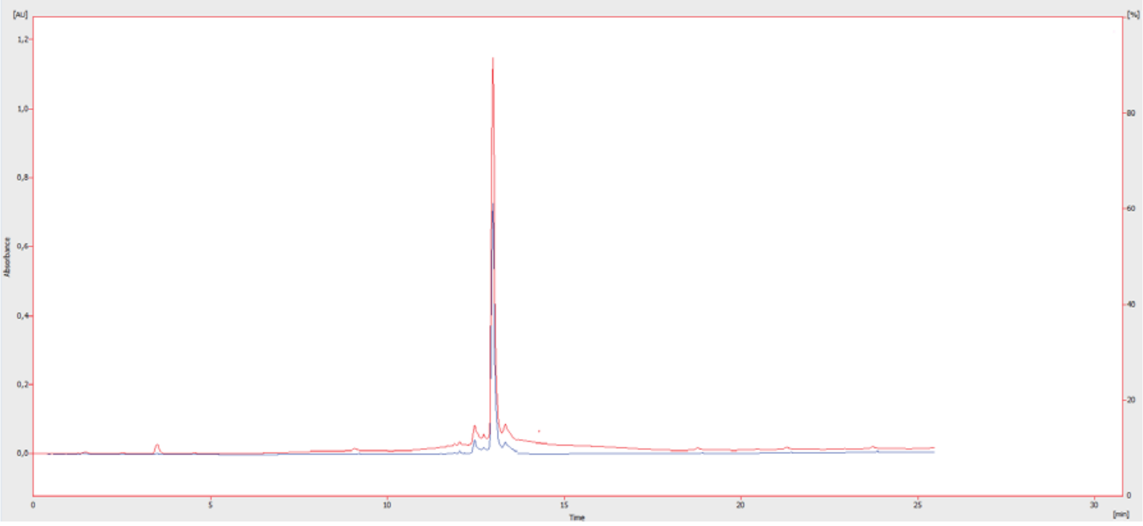

Compound 12a

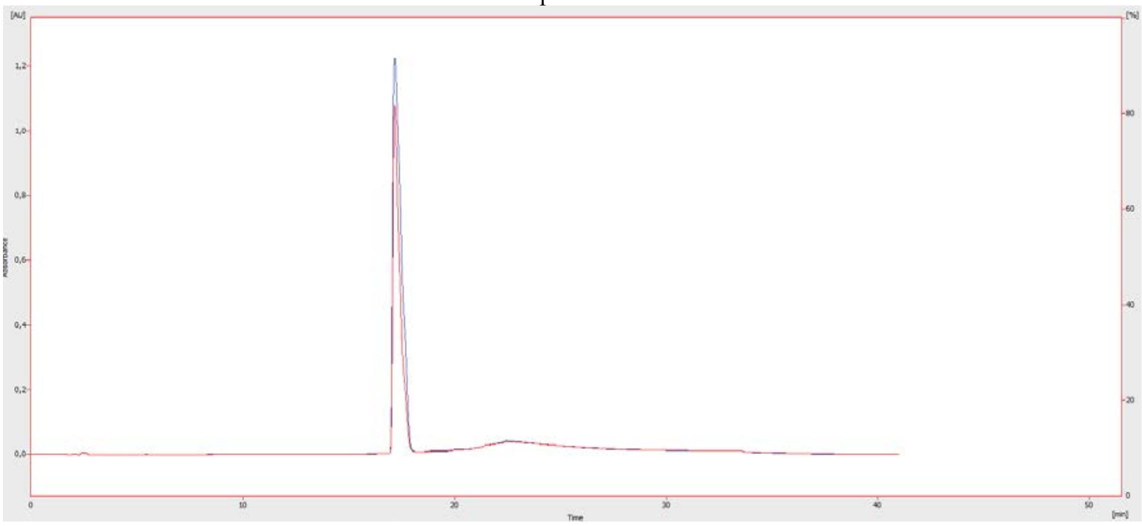

pharmaceutics

MDPI

Figure S1. Analytical HPLC profiles for bola dendrimers **9a**, **10a**, **11a**, **12a**.

Compound 13

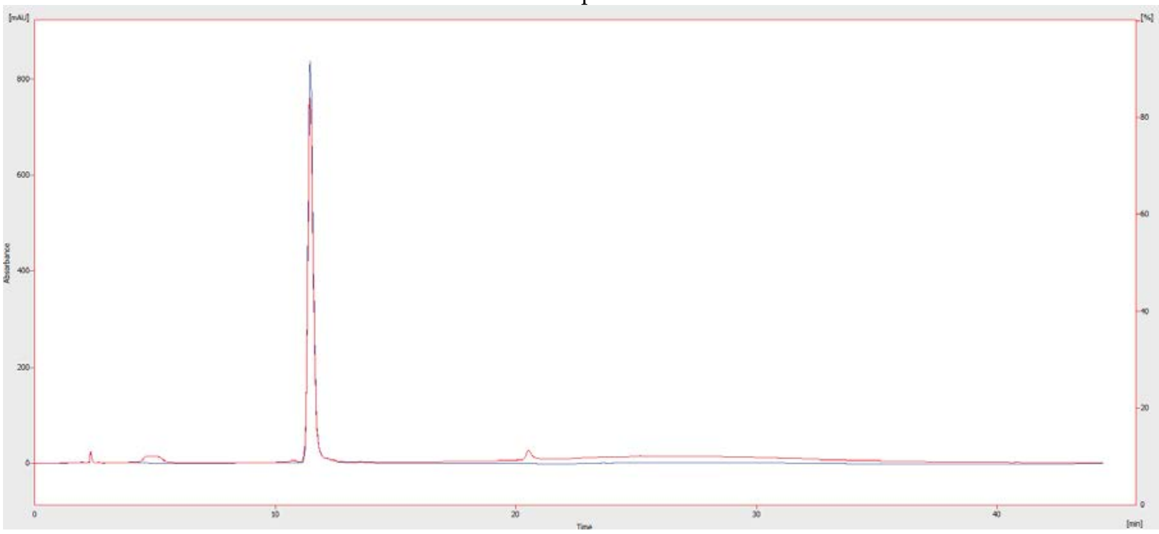

Compound 14

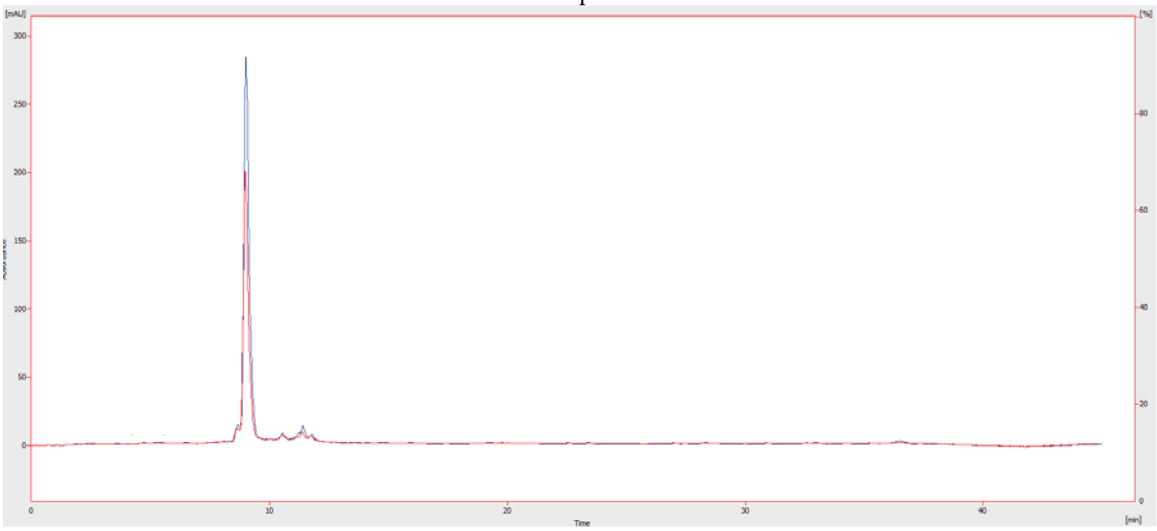

Compound 15

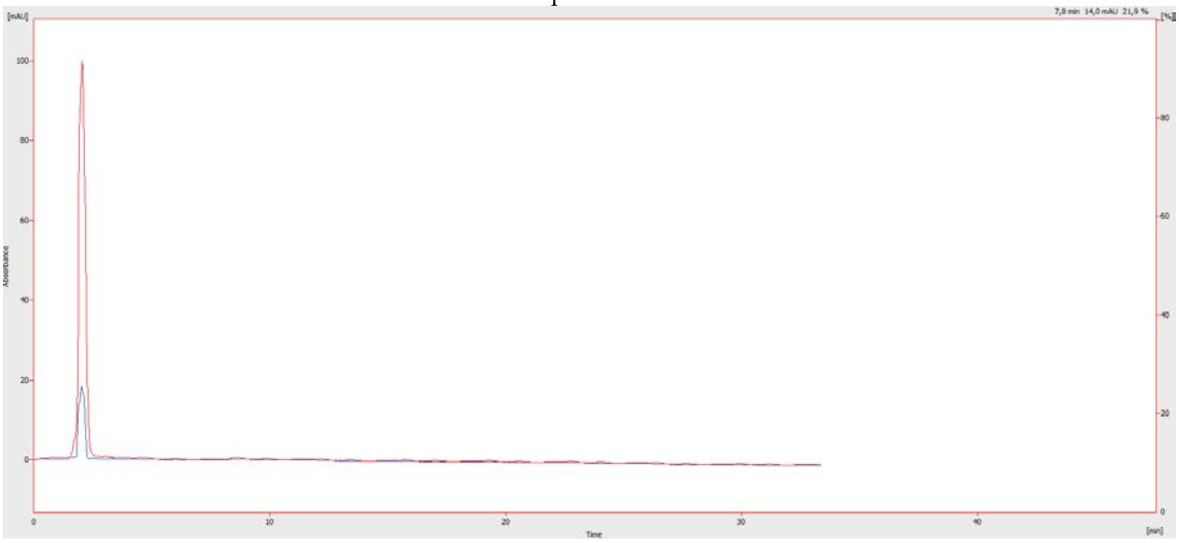

Figure S2. Analytical HPLC profiles for dendrons **13–15**.

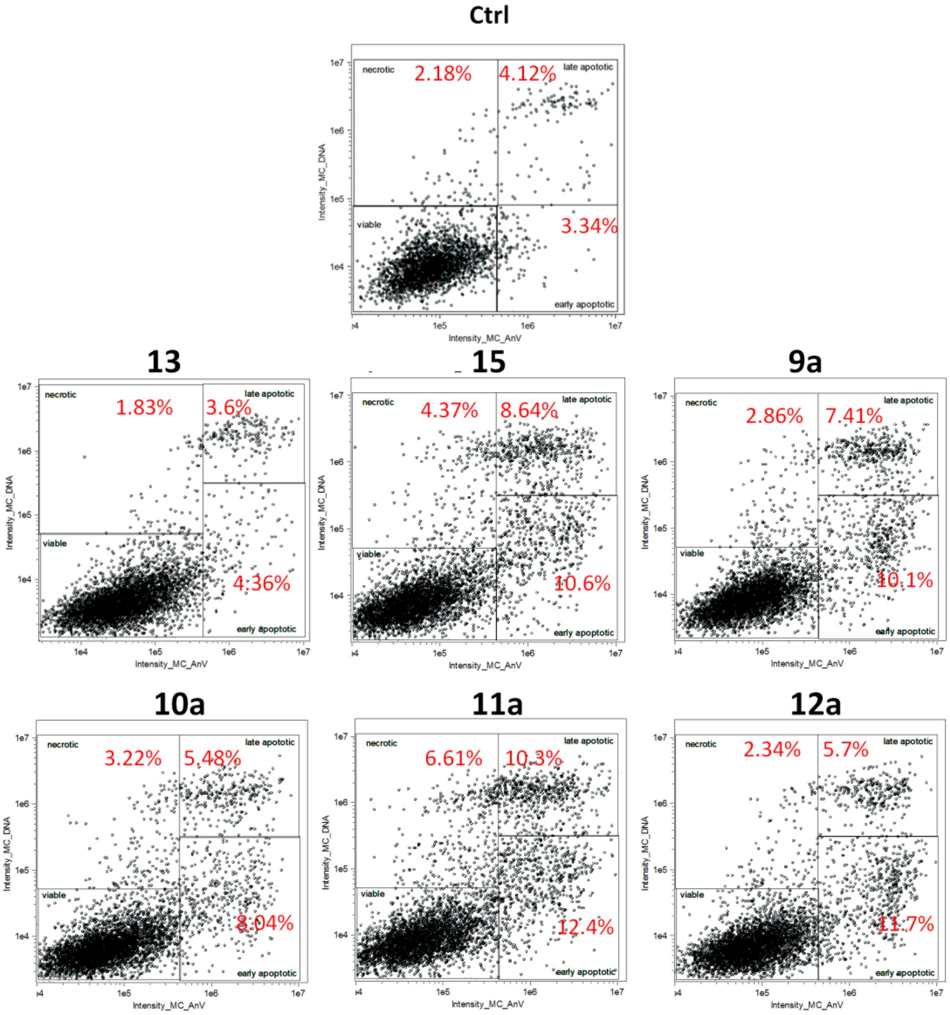

Figure S3. pro-apoptotic effects of ornithine dendrons/dendrimeres in T98G cell populations. Cells were seeded at the density of 2 × 10^5/cm^2. After 24 h of initial incubation, the tested agents were applied at the concentration of 100 ◎ M in the fresh portion of medium for the next 48 h. Subsequently, AnnexinV/Propidium iodide assay was performed (FITC AnnexinV Apoptosis Detection Kit, BD Pharmingen™) using ImageStreamX® cytometer

(Merck Millipore). Data representative for at least 3 independent experiments (*n* > 3) were analyzed with IDEAS® 6.2 software (Merck Millipore).

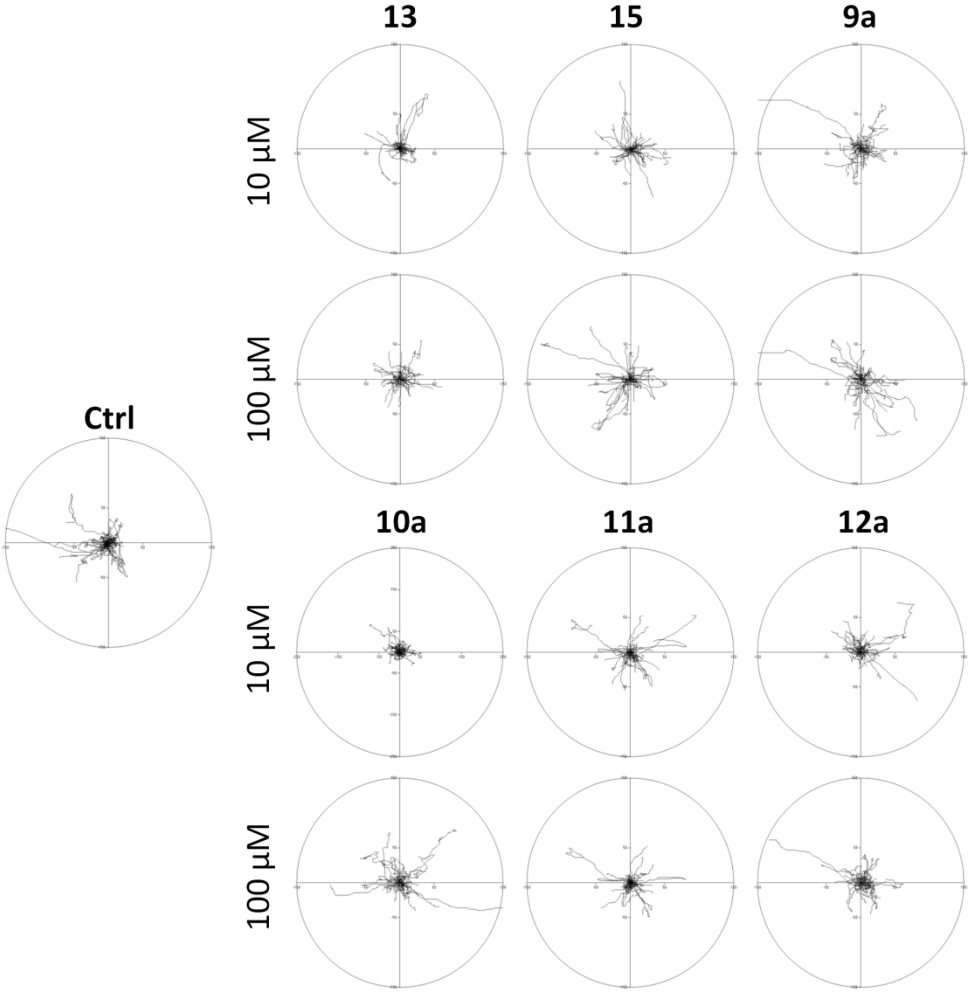

Figure S4. Exemplary plots showing the effect of ornithine dendrons/dendrimers on the motility of T98G cells. Cells were seeded at the density of 2 × 104/cm². After 24 h of initial incubation, the tested agents were applied at the concentration of 10–100 ◎ M and cell movement was registered 48 h afterwards with time-lapse videomicroscopy. Cell trajectories are depicted in circular diagrams (axis scale in µm) drawn with the initial point of each trajectory placed at the origin of the plot (registered for 6 h; *n* > 50). Data representative for at least 3 independent experiments (*n* >3).

pharmaceutics

Synthesis S1

General

All solvents and reagents were of analytical grade and were used without further purification. Coupling reagents *N,N′*-Dicyclohexylcarbodiimide (DCC), *N*-Hydroxysuccinimide (HOSu), *N*-Hydroxybenzotriazol (HOBt), HCl-saturated AcOEt, 4,7,10-trioxa-1,13-tridecanediamine, 4,7,10-trioxa-1,13-tridecanediamine, *O,O′*-bis(2-aminoethyl)polyethylene glycol as well as all solvents were purchased from Sigma (Steinheim, Germany).

Mass spectra were recorded with a Mariner ESI time-of-flight mass spectrometer (PerSeptive Biosystems, Foster City, CA, USA) for the samples prepared in MeOH. The ^{1}H-NMR and ^{13}C-NMR spectra were recorded using a Varian VNMRS 500/125 MHz and 600/150 MHz spectrometers, respectively (Varian, Inc, acquired by Agilent Technologies, Palo Alto, CA, USA) at 500/125 or 400/100 MHz, respectively, using deuterated solvents and TMS as an internal standard. Chemical shifts are reported as δ values in parts per million, and coupling constants are given in hertz. The optical rotations were measured with a JASCO J-1020 digital polarimeter (Ishikawa-machi, Hachioji, Tokyo, Japan). Melting points were recorded on a Köfler hot-stage apparatus (Wagner & Munz, München, Germany) and are uncorrected. Thin layer chromatography (TLC) was performed on aluminum sheets with silica gel 60 F254 from Merck (Darmstadt, Germany). Column chromatography (CC) was carried out using silica gel (230–400 mesh) from Merck or Sephadex LH20 (Darmstadt, Germany or Biosciences, Upsala, Sweden). The TLC spots were visualized by treatment with 1% EtOH solution of ninhydrin and heating.

HPLC analysis for bola was performed with a Knauer HPLC system equipped with a dual wavelength (λ) absorbance detector at 214 and 280 nm (KnauerBerlin, Berlin, Germany). The crude products were purified by preparative HPLC using a C_{18} column, (Bionacom Velocity C-18-LPH) 250 × 212 mm, particle size 10μm, pore diameter of 200 Å, followed by processing by analytical HPLC column (Luna LC-Column, C-8(2)) 150 × 46 mm, particle size 3 μm, pore diameter 100 Å (Bionacom LTD, Coventry, England). The mobile phase consisted of a gradient from 5 to 95% MeOH/H_2O, 0.05% HCl, at a flow rate of 2.0 mL/min (analytical) or 9 mL/min (preparative).

Synthesis S1. Synthesis of Denderimeric Dimers with "Bola" Structure 9a, 10a, 11a, 12a

General Procedure for the Preparation of Peptide Dendrimers in Solution

Peptide dendrimers were obtained using active esters method as shown in Scheme **1**. A divergent approach was used to synthesize desired products based on ornithine monomer using N,N′-Dicyclohexylcarbodiimide (DCC) as a coupling reagent and N-Hydroxysuccinimide (HOSu). The substrate with free amino groups was dissolved in THF, then the excess (1.1 per 1 free amine group) of the corresponding active ester (i.e., Boc-Orn(Boc)-OSu) in THF was added successively. Reaction was carried out at room temperature for 3–5 days and monitored on TLC plates, then the solvent was evaporated to dryness and the post-reaction mixture was dissolved in ethyl acetate and washed with 5% citric acid solution, saturated aqueous $NaHCO_3$ and brine, dried over $MgSO_4$, filtered and evaporated in vacuo. The compounds were purified on Silica Gel or on Sephadex LH-20 and by preparative high performance liquid chromatography (HPLC), then the solvent was removed in vacuo to give the desired compounds as lightly yellow oil. Structures were confirmed on the basis of ^{1}H and ^{13}C NMR.

General Method for the Boc-Deprotection

The tert-βutoxycarbonyl protecting group was removed by treating compounds with 1 M HCl in ethyl acetate (5 mL) for 4 h, followed by removing the solvent in vacuo. The products were washed with diethyl ether and the precipitate was dried in vacuo over P_2O_5.

General method for Fmoc-deprotection

pharmaceutics MDPI

The Fluorenylmethyloxycarbonyl protecting group was removed by treating compounds with 20% piperidine/MeOH solution, followed by removing the solvent in vacuo. The products were washed with diethyl ether and the precipitate was dried in vacuo over P_2O_5.

Synthetic procedures

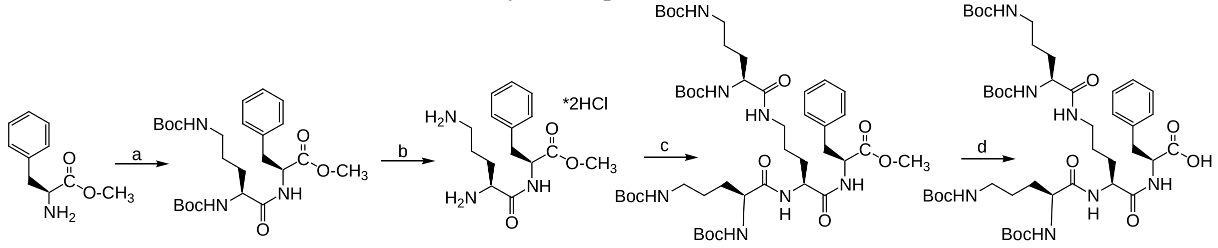

1 2 3 4

Scheme S1. Synthesis of Boc-protected dendron 4. Reagents, conditions (**a**) Boc-Orn(Boc)-OH, DCC/HOSu,THF, 72 h., r.t., yield 90.8%; (**b**) HCl/EtOAc, 4 h, r.t., yield 98.2%; (**c**) Boc-Orn(Boc)-OH, DCC/HOSu,THF, 72 h, r.t., yield: 94.1% ; (**d**) 1M NaOH, 6 h, r.t., yield 89.4%.

Synthetic Procedure for Compound 4

The compound **1** (6.07 g, 12.3 mmol) was obtained by adding to Boc-Orn (Boc)-OH (4.5 g, 13.54 mmol) dissolved in 20 mL THF, HOSu (1.56 g, 13.54 mmol) and DCC (2.79 g, 13.54 mmol) dissolved in 10 mL THF and was stirred for 12 h at room temperature. So prepared active ester was added to phenylalanine methyl ester solution in 10 mL THF (Phe-OMe 2.92 g, 13.54 mmol) and TEA (8.22 g, 81 mmol) and stirred for 72 h at room temperature. The solvent was evaporated and the residue was dissolved in 100 mL of ethyl acetate and was washed 5 times respectively with 5% citric acid solution, saturated aqueous $NaHCO_3$ and brine, dried over MgSO4, filtered and evaporated in vacuo. The compound was purified on Merck Kieselgel silica gel with the mobile phase consisted of 1–5% MeOH/DCM (yield: 90.8%). Subsequently, compound **1** was transformed into its octa-hydrochloride **2** by removing Boc groups with a saturated 1M HCl in AcOEt (yield: 98.2%).

Compound **3** (3.82 g, 4.1 mmol) was obtained by adding to Boc-Orn (Boc)-OH (2.92 g, 8.8 mmol) dissolved in 15 mL THF, HOSu (1 g, 8.8 mmol) and DCC (1.82 g, 8.8 mmol) dissolved in 10 mL THF and was stirred for 12 h at room temperature. So prepared active ester was added to a solution containing compound **2** (1.47 g, 4 mmol) in 10 mL THF and TEA (6.47 g, 64 mmol) and stirred for 72 h at room temperature. The solvent was evaporated and the residue was dissolved in 100 mL of ethyl acetate and was washed 5 times respectively with 5% citric acid solution, saturated aqueous $NaHCO_3$ and brine, dried over MgSO4, filtered and evaporated in vacuo. The compound **3** was purified on Merck Kieselgel silica gel with the mobile phase consisted of 1–5% MeOH/DCM (yield: 94.1%). Subsequently, the phenylalanine methyl ester group was removed from compound **3** in the reaction with 1 M NaOH for 6 h at room temperature to give dendron**4** (yield: 89.4%).

NMR Spectra for Compound 3

^{1}H NMR (600 MHz, CD_3OD), δ: 1.39–1.45 (36H, t-Bu); 1.49–1.81 (m, 12H, CH_2 - β, γ-*Orn*); 3.00–3.07 (m, 5H, CH_2-δ-*Orn* (4H); CH_2-*Phe* (1H)); 3.09–3.19 (m, 2H, CH_2-δ-*Orn* (1H); CH_2-*Phe* (1H)); 3.19–3.26 (m, 1H, CH_2-δ-*Orn*); 3.66 (s, 3H, OMe); 3.95–4.04 (m, 2H, CH α-*Orn*); 4.35–4.41 (m, 1H, *Orn*); 4.64. (dd, J = 8.1, 6.0 Hz, 1H, *Phe*); 7.15–7.30 (m, 5H, *Phe*).

^{13}C NMR (150 MHz, CD_3OD), δ: 26.6 (γ-C-*Orn*); 28.8 [C(**C**H_3)$_3$(Boc)]; 30.6 (β-C-*Orn*); 31.0 (β-C-*Orn*); 38.4 (β-C-*Phe*); 39.6 (δ-C-*Orn*); 40.9 (δ-C-*Orn*); 52.8 (COO**C**H_3 *Phe*); 53.8 (α-C-*Phe*); 55.2 (α-C-*Orn*); 55.7 (α-C-*Orn*); 55.9 (α-C-*Orn*); 79.9 [**C**^{IV}(CH_3)$_3$(Boc)]; 80.6 [**C**^{IV}(CH_3)$_3$(Boc)]; 127.9 (C^4*Phe*); 129.6 ($C^{2,6}$*Phe*); 130.3 ($C^{3,5}$*Phe*); 137.9 (C^1*Phe*); 157.7, 158.5 [**C**=O (Boc)]; 173.3 (O=**C**OCH_3*Phe*); 173.7, 175.0, 175.1 (**C**ONH).

NMR Spectra for Compound 4

^{1}H NMR (600 MHz, CD_3OD), δ: 1.39–1.45 (36H, t-Bu); 1.49–1.81 (m, 12H, CH_2 - β, γ-*Orn*); 3.00–3.07 (m, 5H, CH_2-δ-*Orn* (4H); CH_2-*Phe* (1H)); 3.09–3.19 (m, 2H, CH_2-δ-*Orn* (1H); CH_2-*Phe* (1H)); 3.19–3.26 (m, 1H, CH_2-δ-*Orn*); 3.95–4.04 (m, 2H, CH α-*Orn*); 4.35–4.41 (m, 1H, *Orn*); 4.64. (dd, J = 8.1, 6.0 Hz, 1H, *Phe*); 7.15–7.30 (m, 5H, *Phe*).

^{13}C NMR (150 MHz, CD_3OD), δ: 26.6 (γ-C-Orn); 28.8 [C(**C**H_3)$_3$(Boc)]; 30.6 (β-C-Orn); 31.0 (β-C-Orn); 38.4 (β-C-Phe); 39.6 (δ-C-Orn); 40.9 (δ-C-Orn); 53.8 (α-C-Phe); 55.2 (α-C-Orn); 55.7 (α-C-Orn); 55.9 (α-C-Orn); 79.9 [**C**^{IV}(CH_3)$_3$(Boc)]; 80.6 [**C**^{IV}(CH_3)$_3$(Boc)]; 127.9 (C^4Phe); 129.6 ($C^{2,6}$Phe); 130.3 ($C^{3,5}$Phe); 137.9 (C^1Phe); 157.7, 158.5 [**C**=O (Boc)]; 173.3 (O=**C**OCH_3Phe); 173.7, 175.0, 175.1 (**C**ONH).

Synthesis of the core of the dendrimer (7) and (8)

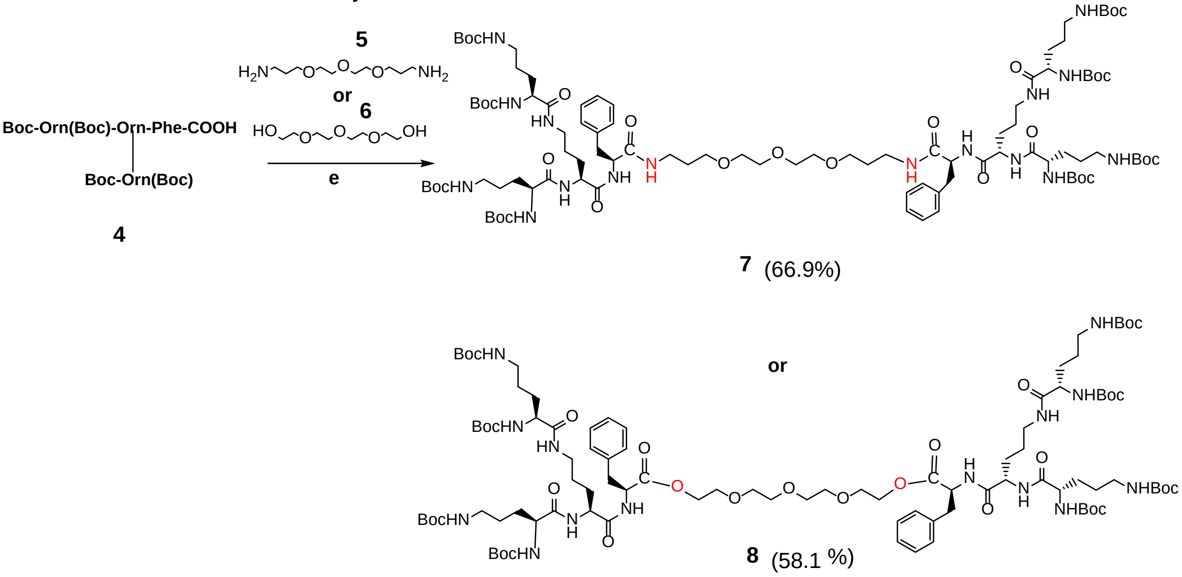

Scheme S2. Synthesis of Boc-protected peptidic bola-dimer **7** connected with amide bonds or Boc-protected bola-dimer **8** connected with ester bonds. Reagents, conditions: (**e**) DCC/HOSu, MeOH/THF, 96 h, r.t. 66.9%, yield for **7** or DCC/DMAP/THF, 96 h, r.t., yield for **8** 58.1%.

Synthetic Procedure for Compound 7

The respective compound **4** (1.6g, 1.76mmol), HOSu (0.2g, 1.76mmol) and DCC (0.39g, 1.89 mmol) were dissolved in THF and stirred overnight. Then the active ester was added to a solution of 4,7,10-trioxa-1,13-tridecnediamine **(5)**(0.19g, 0.88 mmol) and TEA (0.356g, 3.52mmol) in THF and stirred for 96 h at room temperature. The mixture was evaporated and the residue was dissolved in ethyl acetate and washed with 5% citric acid solution, saturated aqueous $NaHCO_3$ and brine, dried over $MgSO_4$, filtered and evaporated in vacuo. The raw dendrimeric compound was purified by molecular filtration on the Sephadex LH-20 packing in MeOH and then by HPLC (yield: 66.9% for **7**). Then the dendrimer was converted to its octahydrochloride by deprotection of Boc groups with HCl-saturated AcOEt to give dendrimer **7a** (yield: 98.6%).

NMR Spectra for Compound 7

^{1}H NMR (600 MHz, MeOD), δ (for half the molecule): 1.36–1.46 (m, 36H, -CH_3 –Boc); 1.47–1.83 (m, 12H, -CH_2*β*, *γ-Orn*); 2.97–3.27 (m, 10H, -CH_2*β-Phe*); HN-CH_2-*linker*, CH_2*δ-Orn*); 3.60–3.67 (m, 6H, -O-CH_2, *linker*); 3.94–4.05 (m, 2H,CH *α-Orn*); 4.30–4.35 (m, 1H,CH*α-Orn*); 4.43–4.48 (m, 1H,CH*α-Phe*); 7.12–7.24(m, 5H,Ar-*Phe*).

^{13}C NMR (150 MHz, CD_3OD), δ (for half the molecule): 26.4 (γ-C-*Orn*); 27.3, 27.5 (γ-C-*Orn*); 28.8 [C(**C**H_3)$_3$(Boc)]; 30.5, 30.7, 30.9 (β-C-*Orn*); 39.0 (β-C-*Phe*); 39.5 (NH-**C**H_2 linker); 39.6 (δ-C-*Orn*); 40.9 (δ-C-*Orn*); 54.2 (*α*-C-*Phe*); 55.6 (*α*-C-*Orn*); 55.9 (*α*-C-*Orn*); 57.1 (*α*-C-*Orn*); 69.9 (O-**C**H_2 linker); 71.1, 71.3 (O-**C**H_2 linker); 79.9 [**C**^{IV}(CH_3)$_3$(Boc)]; 80.6 [**C**^{IV}(CH_3)$_3$(Boc)]; 127.3 (C^4*Phe*); 129.2 ($C^{2,6}$*Phe*); 130.7 ($C^{3,5}$*Phe*); 139.2 (C^1*Phe*); 157.8, 158.5 [**C**=O (Boc)]; 172.8(**C**ONH *Phe*) 174.8, 175.1, 177.2 (**C**ONH).

pharmaceutics

Synthetic Procedure for Compound 8

(According to coupling method described in Bioorg Med Chem Lett. 2016 Aug 1;26(15):3586-9. doi: 10.1016/j.bmcl.2016.06.016. Epub 2016 Jun 8.)

The respective compound **4** (0 78 g, 0 86 mmol), DMAP (0.105g, 0.86 mmol) and DCC (0.185 g, 0.898 mmol) were dissolved in THF and a solution of tetraethylene glycol **(6)** (0.08 g, 0.43 mmol) in THF was added and stirred for 96 h at room temperature. The mixture was evaporated and the residue was dissolved in ethyl acetate and washed with 5% citric acid solution, saturated aqueous $NaHCO_3$ and brine, dried over $MgSO_4$, filtered and evaporated in vacuo. The raw dendrimeric compound was purified by molecular filtration on the Sephadex LH-20 packing in MeOH and then by HPLC (yield: 58.1% for **8**) Then the dendrimer was converted to its octahydrochloride by deprotection of Boc groups with HCl-saturated AcOEt to give dendrimer **8a** (yield: 96.2%).

Functionalization procedures of the dendrimers

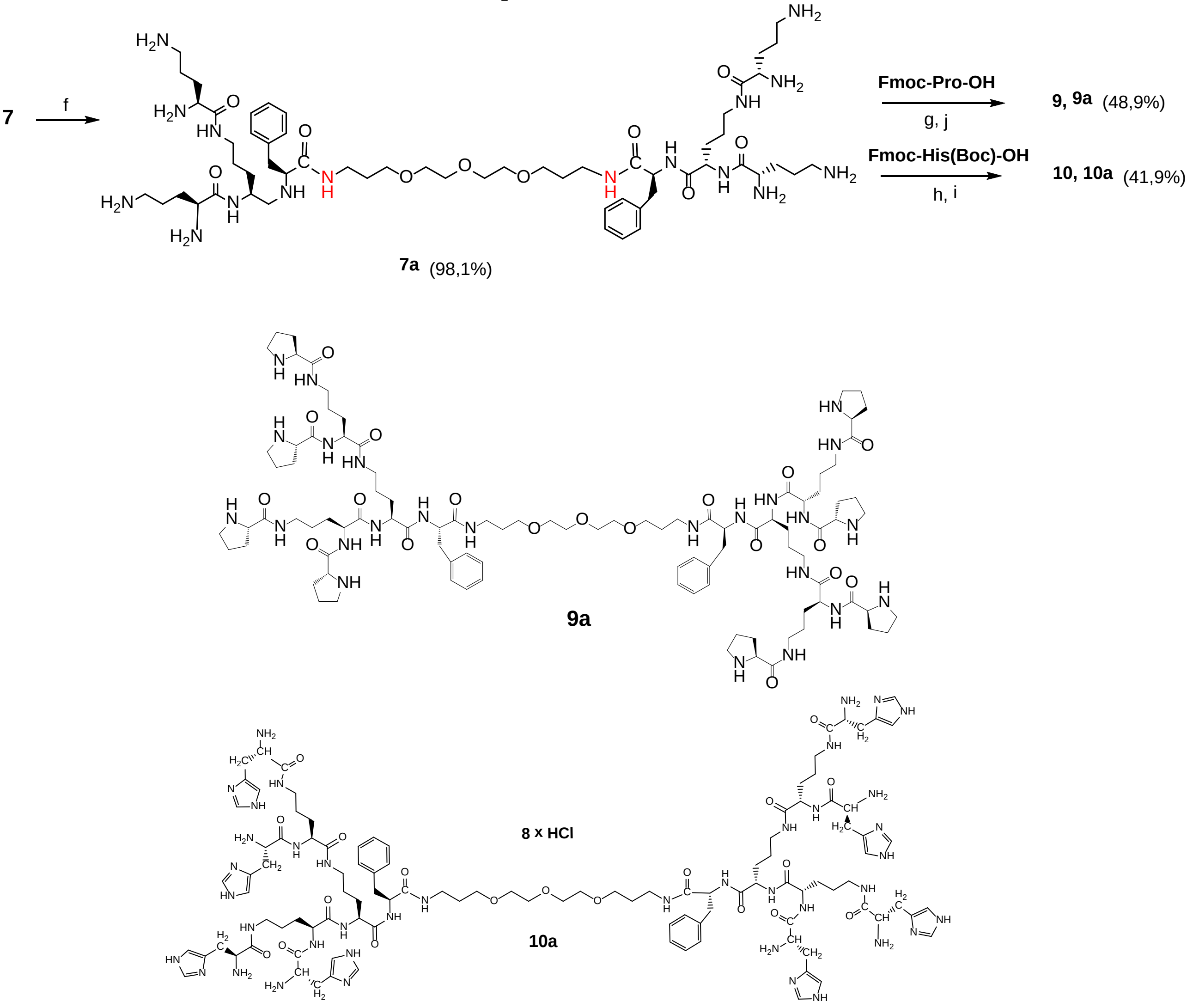

Scheme S3. Synthesis of functionalized bola-dendrimers **9a** and **10a**. Reagents, conditions: (**f**) HCl/EtOAc, 8 h, r.t.; (**g**) Fmoc-Pro-OH, DCC/HOSu,THF, 126 h, r.t.; (**h**) Fmoc-His(Boc)-OH, DCC/HOSu,THF, 126 h., r.t.; (**i**) HCl/EtOAc, 8 h, r.t.; (**j**) 20% piperidine/MeOH 4 h, r.t., yield 48.9% for **9a** and 41.9% for **10a**.

Synthetic Procedure for Proline Decorated Bola-Dendrimer (9a)

Fmoc-Pro-OH (1.3g, 3.84 mmol) HOSu (0.442 g, 3.84 mmol) and DCC (0.824 g, 4 mmol) were dissolved in THF and stirred overnight. Then the active ester was added to a solution of **7a** (0.32 g, 0.16 mmol) and TEA (0.518 g, 5.12 mmol) in THF and stirred for 126 h at room temperature. The mixture was evaporated and the residue was dissolved in ethyl acetate and washed with 5% citric acid solution, saturated aqueous $NaHCO_3$ and brine, dried over $MgSO_4$, filtered and evaporated in vacuo. The raw dendrimeric compound was purified by molecular filtration on the Sephadex LH-20 packing in MeOH and then by HPLC. Then the dendrimer was deprotected of Fmoc groups with 20% piperidine/MeOH solution (yield: 48.9% for **9a**).

NMR Spectra for Compound 9a

^{1}H NMR (600 MHz, MeOD), δ (for half the molecule): 1.50–1.80 (m, CH_2-*β*, *γ-Orn*); 1.70–2.20 (m, CH_2-*Pro*); 2.90–3.06 (m, CH_2-*Pro*, NH-CH_2-*Link*); 3.00–3.20 (m, CH_2-*Phe*); 3.24–3.33 (m, CH_2-*δ-Orn*) 3.43–3.76 (m, O-CH_2-*Link*, CH-*Pro*); 4.26–4.43 (m, CH-*α-Orn*, CH-*α-Phe*); 7.12–7.30 (m, Ar-*Phe*).

pharmaceutics

MDPI

^{13}C NMR (150 MHz, CD_3OD), δ (for half the molecule): 25.3 (γ-C-*Pro*); 26.5 (γ-C-*Orn*); 30.2 (β-C-*Orn*); 31.9 (β-C-*Pro*); 37.0 (NH-**C**H_2linkier); 38.4 (β-C-*Phe*); 38.8 (δ-C-*Orn*); 47.8 (δ-C-*Pro*); 54.0 (α-C-*Phe*); 57.1 (α-C-*Orn*); 61.3 (α-C-*Pro*); 69.6 (O-**C**H_2linkier); 71.0, 71.2 (O-**C**H_2linkier); 127.1 (C^4*Phe*); 128.9 ($C^{2,6}$*Phe*); 130.5 ($C^{3,5}$*Phe*); 139.3 (C^1*Phe*); 173.6 (**C**ONH *Phe*); 176.9, 177.2, 178.9 (**C**ONH).

Synthetic Procedure for Histidine Decorated Bola-Dendrimer (10a)

Fmoc-His(Boc)-OH (2.21 g, 3.84 mmol) HOSu (0.442 g, 3.84 mmol) and DCC (0.824 g, 4 mmol) were dissolved in THF and stirred overnight. Then the active ester was added to a solution of **7a** (0.32 g, 0.16 mmol) and TEA (0.518 g, 5.12 mmol) in THF and stirred for 126 h at room temperature. The mixture was evaporated and the residue was dissolved in ethyl acetate and washed with 5% citric acid solution, saturated aqueous $NaHCO_3$ and brine, dried over $MgSO_4$, filtered and evaporated in vacuo. Then the dendrimer was deprotected of Fmoc groups with 20% piperidine/MeOH solution The raw dendrimeric compound was purified by molecular filtration on the Sephadex LH-20 packing in MeOH and then by HPLC. Then the dendrimer was deprotected of Bocby treating with 1 M HCl in ethyl acetate (5 mL) for 6 h, followed by removing the solvent in vacuo. The product was washed with diethyl ether and the precipitate was *dried* in vacuo over P_2O_5 (yield: 41.9% for **10a**).

NMR Spectra for Compound 10a

^{1}H NMR (600 MHz, MeOD), δ(for half the molecule): 1.38–1.46 (m, CH_3 –Boc); 1.47–1.83 (m, CH_2β, γ-*Orn*); 2.82–3.25 (m, CH_2β-*His*, *δ- Orn*, CH_2β-*Phe*); 3.46–3.67 (m, CH_2, *linker*); 3.93-4.09 (m, CH *α-Orn*); 4.09–4.22 (m, CH*α-Phe*, *α-Orn*); 4.45–4.51 (m, CH-*α -His*); 6.82–6.87 (m, Ar-*His*); 7.11–7.24 (m, Ar-*Phe*); 7.56–7.64 (m, Ar-*His*).

^{13}C NMR (150 MHz, CD_3OD), δ(for half the molecule): 25.7 (γ-C-*Orn*); 27.4, 27.5 (γ-C-*Orn*); 28.8 [C(**C**H_3)$_3$(Boc)]; 30.1, 30.4, 30.9 (β-C-*Orn*); 32.5 (β-C-*His*); 38.3 (δ-C-*Orn*); 39.1 (β-C-*Phe*); 39.7 (NH-**C**H_2linkier); 42.5 (δ-C-*Orn*); 53.9 (α-C-*Phe*); 52.2, 52.8, 55.1, 55.2 (α-C-*His*); 55.9, 56.2 (α-C-*Orn*); 69.8 (O-**C**H_2linkier); 71.2, 71.4 (O-**C**H_2linkier); 79.8 [**C**^{IV}(CH_3)$_3$(Boc)]; 80.5 [**C**^{IV}(CH_3)$_3$(Boc)]; 119.2(**C**=CH *His*); 127.3 (C^4*Phe*); 129.1 ($C^{2,6}$*Phe*); 130.6 ($C^{3,5}$*Phe*); 133.9 (C=**C**H *His*); 136.2 (N-H**C**=N *His*); 139.5 (C^1*Phe*); 158.4 [**C**=O (Boc)]; 171.8 (**C**ONH *Phe*); 172.9, 173.2, 173.3, 173.7, 174.6, 175.5, 177.8 (**C**ONH).

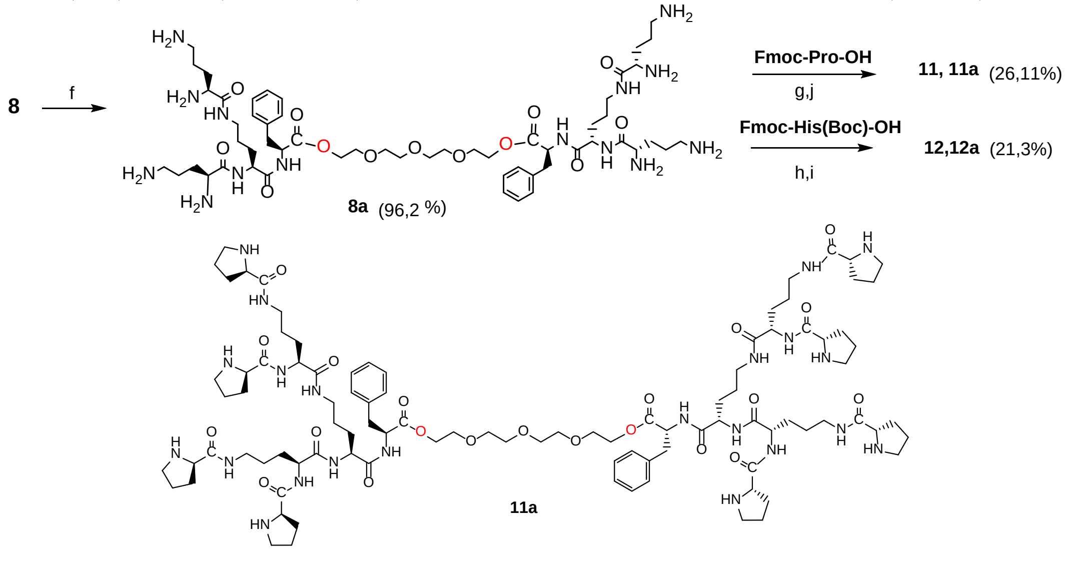

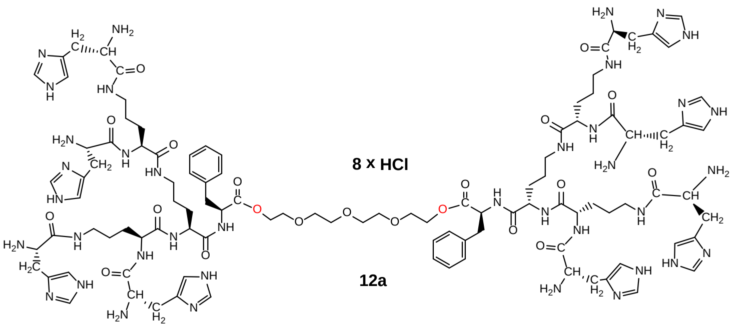

Scheme S4. Synthesis of bola-dendrimers 11a and 12a. Reagents, conditions: (**f**) HCl/EtOAc, 8 h, r.t.; (**g**) Fmoc-Pro-OH, DCC/HOSu,THF, 126 h, r.t.; (**h**) Fmoc-His(Boc)-OH, DCC/HOSu,THF, 126 h, r.t.; (**i**) HCl/EtOAc, 8 h, r.t.; (**j**) 20% piperidine/MeOH, 4 h, r.t.

Synthetic Procedure for Proline Decorated Bola-Dendrimer (11a)

Fmoc-Pro-OH (1.3g, 3.84 mmol) HOSu (0.442 g, 3.84 mmol) and DCC (0.824 g, 4 mmol) were dissolved in THF and stirred overnight. Then the active ester was added to a solution of **8a** (0.3 g, 0.16 mmol) and TEA (0.518 g, 5.12 mmol) in THF and stirred for 126 h at room temperature. The mixture was evaporated and the residue was dissolved in ethyl acetate and washed with 5% citric acid solution, saturated aqueous $NaHCO_3$ and brine, dried over $MgSO_4$, filtered and evaporated in vacuo. The raw dendrimeric compound was purified by molecular filtration on the Sephadex LH-20 packing in MeOH and then by HPLC. Then the dendrimer was deprotected of Fmoc groups with 20% piperidine/MeOH solution (yield: 26.11% for **11a**).

NMR Spectra for Compound 11a

^{1}H NMR (600 MHz, MeOD), δ (for half the molecule): 1.45–1.58 (m, CH_2-*γ-Orn*); 1.67–2.18 (m, *β*, -*Orn*, CH_2-*Pro*);2.89–2.99 (m, CH_2-*Pro*); 2.98–3.27 (m, CH_2-*Phe*, CH_2-*δ-Orn*); 3.53–3.77 (m, O-CH_2-*Link*, CH-*Pro*); 4.16–4.47 (m, CH-*α-Orn*, CH-*α*-*Phe*); 7.11–7.30 (m, Ar-*Phe*).

^{13}C NMR (150 MHz, CD_3OD), δ (for half the molecule): 26.8 (γ-C-*Pro*); 28.7 (γ-C-*Orn*); 30.4 (β-C-*Orn*); 32.1 (β-C-*Pro*); 38.3 (δ-C-*Orn*); 39.1 (*β*-C-*Phe*); 39.7 (δ-C-*Orn*); 48.0 (δ-C-*Pro*); 54.3 (*α*-C-*Orn*); 57.4 (*α*-C-*Phe*); 61.6, 61.7 (*α*-C-*Pro*); 71.3, 71.5 (O-**C**H_2 linkier); 127.3 (C^4*Phe*); 129.2 ($C^{2,6}$*Phe*); 130.4 ($C^{3,5}$*Phe*); 139.5 (C^1*Phe*); 173.3 (**C**ONH *Phe*); 174.2, 176.8, 177.0, 177.6 (**C**ONH).

Synthetic Procedure for Histidine Decorated Bola-Dendrimer (12a)

Fmoc-His(Boc)-OH (2.21 g, 3.84 mmol) HOSu (0.442 g, 3.84 mmol) and DCC (0.824 g, 4 mmol) were dissolved in THF and stirred overnight. Then the active ester was added to a solution of **8a** (0.3 g, 0.16 mmol) and TEA (0.518 g, 5.12 mmol) in THF and stirred for 126 h at room temperature. The mixture was evaporated and the residue was dissolved in ethyl acetate and washed with 5% citric acid solution, saturated aqueous $NaHCO_3$ and brine, dried over $MgSO_4$, filtered and *evaporated* in vacuo. Then the dendrimer was deprotected of Fmoc groups with 20% piperidine/MeOH solution The raw dendrimeric compound was purified by molecular filtration on the Sephadex LH-20 packing in MeOH and then by HPLC. Then the dendrimer was deprotected of Bocby treating with 1M HCl in ethyl acetate (5 mL) for 6 h, followed by removing the solvent in vacuo. The product was washed with diethyl ether and the precipitate was *dried in vacuo* over P_2O_5 (yield: 21.3% for **12a**).

NMR Spectra for Compound 12a

^{1}H NMR (600 MHz, MeOD), δ(for half the molecule):1.12–1.83 (m, CH_2 **-** β, γ-*Orn*);3.31–3.38 (m, CH_2*β-His*, *δ- Orn*, CH_2β-*Phe*); 3.40–3.47 (m, CH *α-Orn*); 3.48–3.57 (m, CH_2- *δ- Orn*);3.59–3.61 (m, CH_2-*linker*); 3.63–3.66 (m, CH *α-Orn*); 3.67–3.72 (m, CH_2-*linker*); 4.21–4.28 (m, CH-*α*-*His*);7.36–7.40 (m, Ar-*Phe*); 7.52–7.55 (m, Ar-*His*); 8.93–8.96 (m, Ar-*His*).

^{13}C NMR (150 MHz, CD_3OD), selected signals δ: 25.4 (γ-C-*Orn*); 26.0, 26.6 (β-C-*Orn*); 27.7 (δ-C-*Orn*); 44.1, 45.9 (β-C-*His*); 48.1 (β-C-*Phe*); 49.8, 50.2 (δ-C-*Orn*); 53.4 (α-C-*Phe*); 52.1, 55.5 (α-C-*Orn*); 60.5, 61.9 (α-C-*His*); 71.0, 71.3, 73.4 (O-**C**H_2linkier); 119.7 (**C**=CH *His*); 127.2 (C^4*Phe*); 128.7 ($C^{2,6}$*Phe*); 129.7 ($C^{3,5}$*Phe*); 134.5 (C=**C**H *His*); 135.6 (N-H**C**=N *His*); 137.8 (C^1*Phe*); 167.9 (**C**ONH *Phe*); 172.2, 173.2, 174.2, 177.1, 177.2, 177.4 (**C**ONH).

pharmaceutics

MDPI

Synthesis S2

Synthesis of peptide dendrons decorated with proline, histidine or arginine

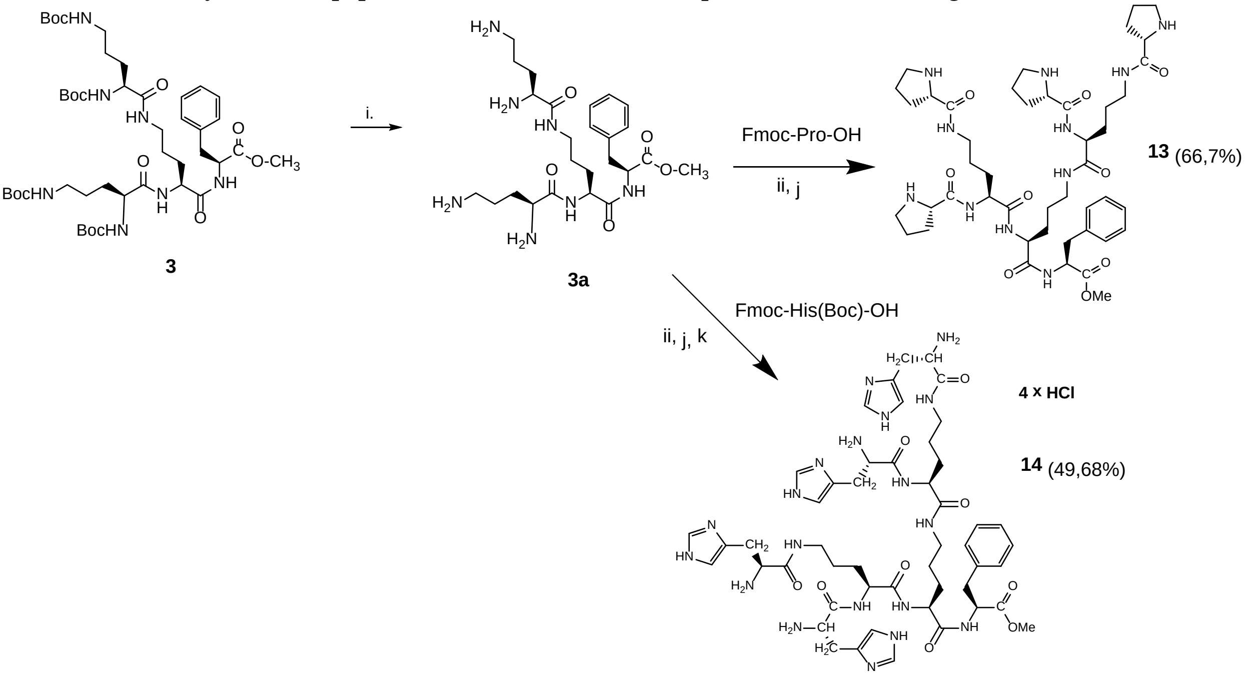

Scheme S5. Synthesis of peptide dendrons 13 and 14 in solution: (**i**) HCl/EtOAc, 8 h, r.t.; (**ii**) Fmoc-Pro-OH, DCC/HOSu,THF, 126 h, r. t.; or Fmoc-His(Boc)-OH, DCC/HOSu,THF, 126 h, r.t.; (**j**) 20% piperidine/MeOH; (**k**) HCl/EtOAc 8 h, r.t.

Synthetic Procedure for Proline Decorated Dendron (13)

Fmoc-Pro-OH (1.34 g, 3.96 mmol) HOSu (0.46 g, 3.96 mmol) and DCC (0.91 g, 4.4 mmol) were dissolved in THF and stirred overnight. Then the active ester was added to a solution of **3a** (0.3 g, 0.33 mmol) and TEA (0.801 g, 7.92 mmol) in THF and stirred for 96 h at room temperature. The mixture was evaporated and the residue was dissolved in ethyl acetate and washed with 5% citric acid solution, saturated aqueous $NaHCO_3$ and brine, dried over $MgSO_4$, filtered and evaporated in vacuo. The raw dendron was purified by preparative HPLC using a C_{18} column, 250 × 21.20 mm, particle size 15 µm and a pore diameter of 200 Å. The mobile phase consisted of a gradient from 5 to 95% MeOH/H_2O, 0.05% HCl, at a flow rate of 3.0 mL/min. Then the dendron was deprotected of Fmoc groups with 20% piperidine/MeOH solution to give compound **13**, yield: 66.7%

NMR Spectra for Compound 13

^{1}H NMR (600 MHz, CD_3OD), δ: 1.43–2.26 (m, CH_2-*β*, *γ-Orn*, CH_2-*Pro*),2.83–3.27 (m, CH_2-*Phe*, CH_2-*δ-Orn*), 3.52–3,68 (m, CH_2-*Pro,* OMe), 4.22–4.47 (m, CH-*α-Orn,* CH-*α-Pro*),4.57–4.68 (m, CH-*Phe*), 7.10–7.31 (m, Ar-*Phe*).

^{13}C NMR (150 MHz, CD_3OD), selected signals δ: 25.6 (γ-C-*Pro*); 26.5 (γ-C-*Orn*); 30.3 (β-C-*Orn*); 31.1 (β-C-*Pro*); 38.4 (β-C-*Phe*); 39.8 (δ-C-*Orn*); 48.0, 48.3 (δ-C-*Pro*); 52.7(α-C-*Phe*); 54.4, 55.3 (α-C-*Orn*); 65.0, 66.3 (α-C-*Pro*); 127.9 (C^4*Phe*); 129.5 ($C^{2,6}$*Phe*); 130.4 ($C^{3,5}$*Phe*); 138.1 (C^1*Phe*); 173.2, 173.3, 174.2, 175.5 (**C**ONH).

Synthetic Procedure for Histidine Decorated Dendron (14)

Fmoc-His(Boc)-OH (3.3 g, 5.73 mmol) HOSu (0.6 g, 5.73 mmol) and DCC (1.29 g, 5.73 mmol) were dissolved in THF and stirred overnight. Then the active ester was added to a solution of **3a** (0.44 g, 0.48 mmol) and TEA (1.16 g, 11.46 mmol) in THF and stirred for 96 h at room temperature. The mixture was evaporated and the residue was dissolved in ethyl acetate and washed with 5% citric acid

solution, saturated aqueous $NaHCO_3$ and brine, dried over $MgSO_4$, filtered and evaporated in vacuo. The raw dendron was purified by preparative HPLC using a C_{18} column, 250 × 21.20 mm, particle size 15 μm and a pore diameter of 300 Å. The mobile phase consisted of a gradient from 5 to 95% MeOH/H_2O, 0.05% HCl, at a flow rate of 3.0 mL/min. Then the dendron was deprotected of Fmoc groups with 20% piperidine/MeOH solution to give compound **14**, yield: 49.68%

NMR Spectra for Compound 14

^{1}H NMR (600 MHz, MeOD), δ (for half the molecule):1.12–1.83 (m, CH_2 **-** β, γ-*Orn*);3.31–3.38 (m, CH_2*β-His*, *δ- Orn*, CH_2β-*Phe*); 3.40–3.47 (m, CH *α-Orn*); 3.48–3.57 (m, CH_2- *δ- Orn*);3.63–3.66 (m, CH *α-Orn*); 4.21–4.28 (m, CH-*α*-*His*);7.36–7.40 (m, Ar-*Phe*); 7.52–7.55 (m, Ar-*His*); 8.93–8.96 (m, Ar-*His*).

^{13}C NMR (150 MHz, CD_3OD), selected signals δ: 25.4 (γ-C-*Orn*); 26.0, 26.6 (β-C-*Orn*); 27.7 (δ-C-*Orn*); 44.1, 45.9 (β-C-*His*); 48.1 (β-C-*Phe*); 49.8, 50.2 (δ-C-*Orn*); 53.4 (*α*-C-*Phe*); 52.1, 55.5 (*α*-C-*Orn*); 60.5, 61.9 (*α*-C-*His*); 119.7 (**C**=CH *His*); 127.2 (C^4*Phe*); 128.7 ($C^{2,6}$*Phe*); 129.7 ($C^{3,5}$*Phe*); 134.5 (C=**C**H *His*); 135.6 (N-H**C**=N *His*); 137.8 (C^1*Phe*); 167.9 (**C**ONH *Phe*); 172.2, 173.2, 174.2, 177.2 (**C**ONH).

Synthetic Procedure for Arginine Decorated Dendron (15)

Fmoc-protected Phe-TentaGel PHB resin (resin preloaded with phenylalanine) (2 g; 0.5 mmol/g) was swollen in DMF for 4 h. The Fmoc group was removed using two 5 min treatments with 2:8 piperidine/DMF, and washed thoroughly with DMF. Once drained, the resin was acylated with a solution containing Fmoc-Lys(Fmoc)-OH (0.5907 g; 1 mmol), [2-(7-Aza-1H-benzotriazole-1-yl)-1, 1, 3, 3-tetramethyluronium hexafluorophosphate] (HATU; 0.7605 g; 1.86 mmol), and N, N-Diisopropylethylamine (DIPEA; 0.736 mL, 4.23 mmol) in anhydrous DMF for 4 h at RT. After being drained and washed with DMF, the Fmoc group was removed, as previously described. After draining the resin, the acylation procedure was repeated for 6 h with a solution containing Fmoc-Lys(2-Cl-Z)-OH (1.0740 g; 2 mmol), HATU (1.5209 g; 4 mmol), and DIPEA (1472 mL, 8,45 mmol) in anhydrous DMF. After being drained and washed with DMF, the Fmoc group was removed and the resin was washed with DMF. The last acylation procedure was performed for 8 h with a solution containing Fmoc-Arg(NO_2)-OH (0.8828 g; 2 mmol), HATU (15.209 g; 4 mmol), and DIPEA (1472 mL, 8.45 mmol) in anhydrous DMF. After being drained and washed with DMF, the Fmoc group was removed, as previously described. After being drained and washed with DMF, the peptide-resin was deprotected and released by treatment with a TFA/H_2O (9:1) solution at RT for 4 h. The resin was filtered off and washed with ethyl acetate. The volatiles were then removed in vacuo, and the crude product was precipitated twice with diethyl ether. The crude product **15** was purified by preparative HPLC using a C18 column, 250 × 21.20 mm, particle size 15 μm and a pore diameter of 300 Å. The mobile phase consisted of a gradient from 5% to 95% MeOH/H_2O, 0.05% HCl, at a flow rate of 3.0 mL/min. Final yield: 51.85%. The synthetic pathway for Dendron **15** is shown in Scheme S6.

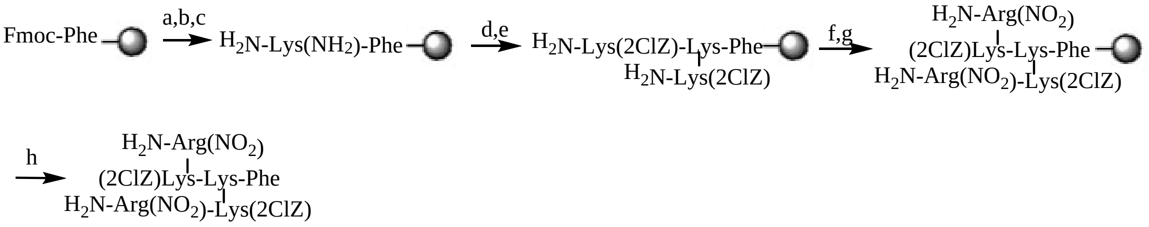

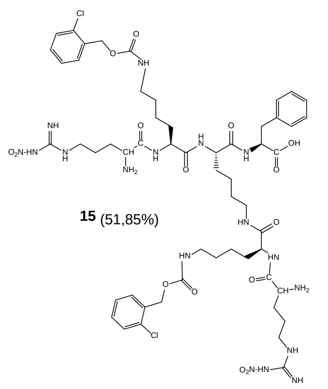

15 (51,85%)

Scheme S6. Synthesis of peptide dendron 15 on solid suport: (**a**) 20% piperidine/DMF, (**b**) Fmoc-Lys(Fmoc)-OH, HATU, DIPEA, (**c**) 20% piperidine/DMF, (**d**) Fmoc-Lys(2-Cl-Z)-OH, HATU, DIPEA (**e**) 20% piperidine/DMF, (**f**) Fmoc-Arg(NO2)-OH, HATU, DIPEA (**g**) 20% piperidine/DMF (**h**) 90% TFA/H2O

NMR Spectra for compound 15

^{1}H NMR (600 MHz, CD_3OD), δ: 1.36–2.11 (m, β, γ, δ CH_2-*Lys,* γ- CH_2-*Arg*), 2.33–2.40 (m, β CH_2-*Arg*), 2.92–3.27 (m, CH_2-*Phe,* ε CH_2-*Lys,*δ-CH_2-*Arg*), 4.10–4.21 (m, α CH-*Lys,* CH-*Fmoc*), 4.34–4.41 (m, α CH-*Phe,* CH_2-*Fmoc*), 4.63–4.676 (m, α CH-*Arg*), 5.05–5.13 (m, CH_2-*2-Cl-Z*), 7.16–7.42 (m, Ar-*Phe,* Ar-*Fmoc,* Ar-*2-Cl-Z*), 7.58–7.65 (m,Ar-*2-Cl-Z*), 7.73–7.78 (m, Ar-*Fmoc*)^{13}C NMR (150 MHz, CD_3OD) selected signals δ: 24.0, 24.3, 24.7, 24.8, 28.9, 29.8, 30.1, 30.2, 30.4, 31.3, 32.1, 38.3, 41.5, 41.8, 48.4, 53.5, 55.8, 64.6, 67.9, 68.2, 120.9, 126.2, 128.1, 128.2, 128.8, 130.3, 130.4, 134.1, 135.9, 138.2, 142.6, 145.1, 145.3, 158.4, 158.6, 161.0, 174.5, 175.2, 176.6, 177.2.

Table S1. Chemical data

Compound	Structure	Formula/MW/m.p.[1)]
**9a**	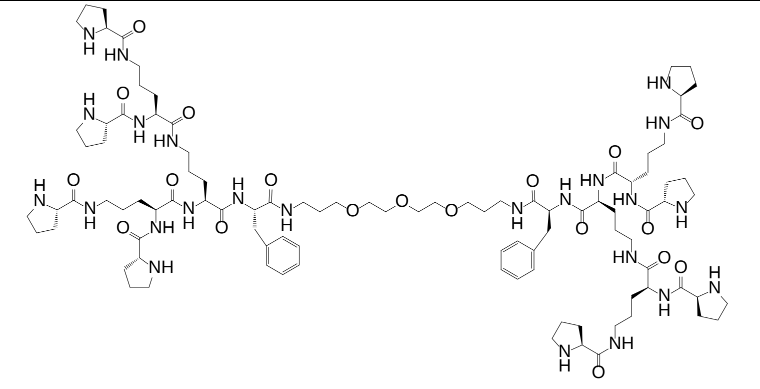	$C_{96}H_{154}N_{24}O_{19}$<br>MW= 1976,44 g/mol<br>134.4 °C
**10a**	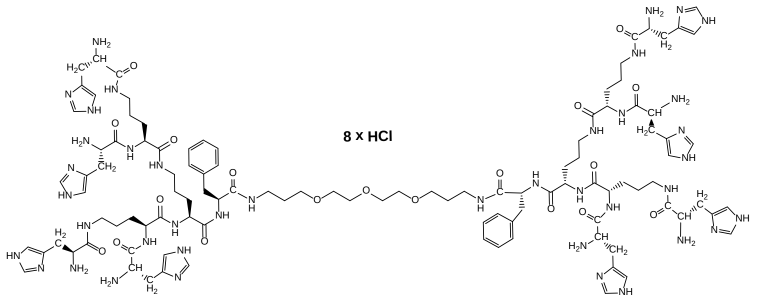	$C_{104}H_{162}N_{40}O_{19}Cl_8$<br>MW = 2296,64 g/mol<br>129.3 °C
**11a**	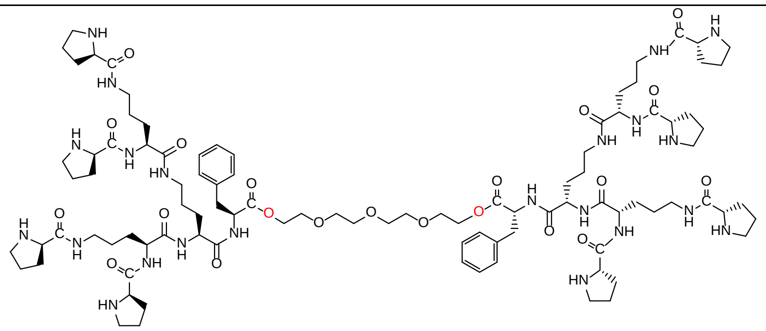	$C_{96}H_{152}N_{22}O_{21}$<br>MW = 1950,41 g/mol<br>178.2 °C
**12a**	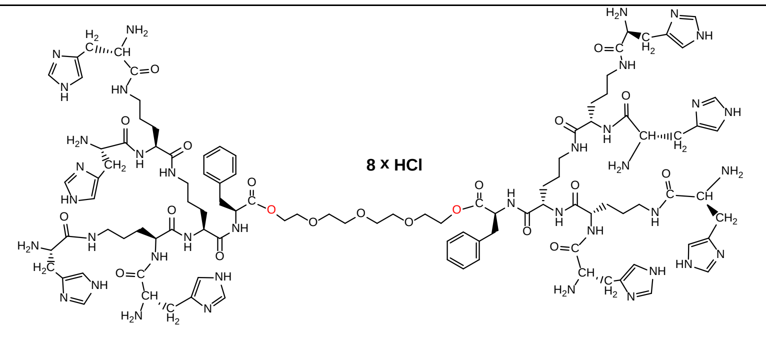	$C_{104}H_{160}N_{38}O_{21}Cl_8$<br>MW = 2270,61 g/mol<br>177.7 °C

**13**	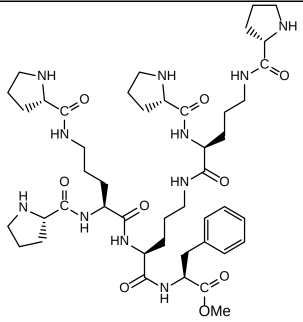	$C_{45}H_{71}N_{11}O_9$<br>MW = 910,13 g/mol<br>139.2 °C
**14**	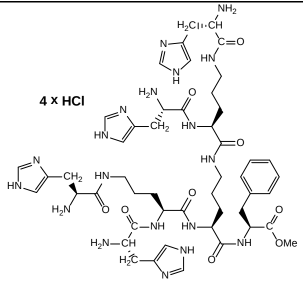	$C_{49}H_{75}N_{19}O_9Cl_4$<br>MW = 1216,23 g/mol<br>131.7 °C
**15**	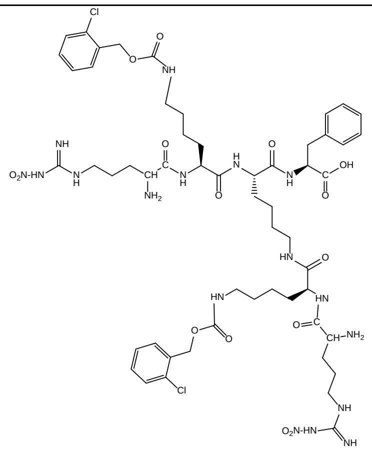	$C_{55}H_{79}N_{17}O_{15}Cl_2$<br>2 × TFA<br>MW= 1289,2 g/mol<br>164.3 °C

[1)] Melting points were recorded on a Köfler hot-stage apparatus and are uncorrected.
